# The Burkholderia cenocepacia Type VI Secretion System Effector TecA Is a Virulence Factor in Mouse Models of Lung Infection

**DOI:** 10.1128/mBio.02098-21

**Published:** 2021-09-28

**Authors:** Nicole A. Loeven, Andrew I. Perault, Peggy A. Cotter, Craig A. Hodges, Joseph D. Schwartzman, Thomas H. Hampton, James B. Bliska

**Affiliations:** a Department of Microbiology and Immunology, Geisel School of Medicine at Dartmouth College, Hanover, New Hampshire, USA; b University of North Carolina at Chapel Hill School of Medicine, Chapel Hill, North Carolina, USA; c Department of Genetics and Genome Sciences, Case Western Reserve University, Cleveland, Ohio, USA; Yale University School of Medicine

**Keywords:** *Burkholderia cenocepacia*, lung infection, type VI secretion

## Abstract

Burkholderia cenocepacia is a member of the Burkholderia cepacia complex (Bcc), a group of bacteria with members responsible for causing lung infections in cystic fibrosis (CF) patients. The most severe outcome of Bcc infection in CF patients is cepacia syndrome, a disease characterized by necrotizing pneumonia with bacteremia and sepsis. B. cenocepacia is strongly associated with cepacia syndrome, making it one of the most virulent members of the Bcc. Mechanisms underlying the pathogenesis of B. cenocepacia in lung infections and cepacia syndrome remain to be uncovered. B. cenocepacia is primarily an intracellular pathogen and encodes the type VI secretion system (T6SS) effector TecA, which is translocated into host phagocytes. TecA is a deamidase that inactivates multiple Rho GTPases, including RhoA. Inactivation of RhoA by TecA triggers assembly of the pyrin inflammasome, leading to secretion of proinflammatory cytokines, such as interleukin-1β, from macrophages. Previous work with the B. cenocepacia clinical isolate J2315 showed that TecA increases immunopathology during acute lung infection in C57BL/6 mice and suggested that this effector acts as a virulence factor by triggering assembly of the pyrin inflammasome. Here, we extend these results using a second B. cenocepacia clinical isolate, AU1054, to demonstrate that TecA exacerbates weight loss and lethality during lung infection in C57BL/6 mice and mice engineered to have a CF genotype. Unexpectedly, pyrin was dispensable for TecA virulence activity in both mouse infection models. Our findings establish that TecA is a B. cenocepacia virulence factor that exacerbates lung inflammation, weight loss, and lethality in mouse infection models.

## INTRODUCTION

Burkholderia cenocepacia belongs to a group of Gram-negative bacteria known as the Burkholderia cepacia complex (Bcc) that exist in the environment and are notorious for causing opportunistic lung infections in immunocompromised patients (1, 2). B. cenocepacia infections are most commonly diagnosed in cystic fibrosis (CF) patients; however, other immunocompromised individuals are also susceptible (2). Bcc infections are difficult to eradicate due to intrinsic antibiotic resistance (1). Patients may present with asymptomatic, chronic, or severe infection. A severe and rapid onset form of this disease, cepacia syndrome, is characterized by necrotizing pneumonia and bacteremia, which can lead to sepsis and is often fatal in CF patients. Of the species in the Bcc, B. cenocepacia accounts for ∼45% of isolates from CF patients and is most often associated with severe disease and cepacia syndrome (3).

CF is a disease resulting from mutations in the cystic fibrosis transmembrane conductance regulator (CFTR) gene. These mutations decrease or prevent chloride ion transport, causing dehydration of the mucosal layer of the lung and gut epithelium (4). The most common mutation, present in at least one allele of *CFTR* in over 90% of CF patients, is the deletion of phenylalanine 508 (F508del) (5). In the CF lung, dehydrated mucus prevents proper mucociliary clearance, creating a prime environment for colonization and infection predominantly by bacterial pathogens. *CFTR*, however, is not expressed only in epithelial cells. Innate immune cells also express low levels of *CFTR*, and it has been shown that lack of proper chloride ion transport also affects their activities (6). CF neutrophils, for example, display a variety of defective responses that cause them to be hyperinflammatory yet less bactericidal (7–9). This leads to frequent infections, predominantly bacterial, starting at a young age that begins the cycle of obstruction, infection, inflammation, and exacerbation. Key bacterial pathogens in CF include Pseudomonas aeruginosa, Staphylococcus aureus, Haemophilus influenzae, Stenotrophomonas maltophilia, *Achromobacter*, and members of the Bcc (3). Patients are often colonized with bacteria for life, resulting in chronic inflammation and cumulative lung fibrosis leading to lung disease, the major cause of death of CF patients (4, 10).

B. cenocepacia is primarily but not strictly an intracellular pathogen that encodes an arsenal of virulence factors (1, 2, 11). *In situ* imaging of infected human and mouse lung tissue indicates that B. cenocepacia resides primarily in phagocytic cells (12, 13), but other cells may be infected, including the epithelium (14). One factor implicated in the pathogenesis of B. cenocepacia is a type VI secretion system (T6SS), designated T6SS-1 (15–17). T6SS-1 can deliver effectors into host cells (17) or other bacteria (15, 16) that come into contact with B. cenocepacia. A transposon mutagenesis screen identified T6SS-1 as an important virulence determinant in a chronic rat lung infection model (17, 18). Studies of macrophages infected *in vitro* showed that the T6SS-1 is used by intracellular B. cenocepacia to inactivate the host GTPases Rac1 and Cdc42 (17). Inactivation of these GTPases in macrophages is associated with reduced phagocytosis and diminished assembly of NADPH oxidase complexes on phagosomes harboring B. cenocepacia (17). Additionally, T6SS-1 was shown to be required for B. cenocepacia to trigger assembly of the pyrin inflammasome in macrophages (19). Canonical inflammasome assembly leads to caspase-1 processing and subsequent cleavage of gasdermin-D (GSDMD), pro-interleukin-1β (pro-IL-1β), and pro-IL-18. The mature forms of these proinflammatory cytokines as well as IL-1α are subsequently released through GSDMD pores, and the infected cell may undergo pyroptosis (20–22). Xu et al. (22) discovered that inactivation of Rho GTPases (RhoA/B/C) in macrophages infected with B. cenocepacia in a T6SS-1-dependent manner is responsible for activation of the pyrin inflammasome. This work established that pyrin, encoded by the *Mefv* gene, is an intracellular inflammasome sensor that specifically and indirectly senses inactivation of RhoA/B/C (here referred to as RhoA) by bacterial effectors and toxins (22–24). Pyrin is the only inflammasome sensor that is preferentially expressed in phagocytes such as neutrophils, monocytes, and activated macrophages (25).

TecA (T6SS effector protein affecting cytoskeletal architecture) was subsequently identified as an enzyme encoded by B. cenocepacia that is injected into host phagocytes and inactivates RhoA and Rac1 (26). Additionally, Aubert et al. determined that TecA deamidates asparagine-41 of RhoA, triggering pyrin activation and inflammasome assembly (26). Thus, pyrin inflammasome assembly in response to inactivation of RhoA by TecA is an effector-triggered immune response that is mechanistically similar to the “guard hypothesis” in plant immunity (26). This work also identified *tecA* genes in other B. cenocepacia strains and *tecA*-like gene sequences in other bacteria (26). A putative protein structure for TecA was generated that led to a prediction that cysteine-41 comprises part of a catalytic triad (26). A codon change mutation of cysteine-41 to alanine (C41A) in *tecA* resulted in loss of catalytic function for TecA and prevented pyrin inflammasome activation in B. cenocepacia-infected macrophages (26).

Studies using acute lung infection models in mice have been carried out with the B. cenocepacia strain J2315 (BcJ2315) to understand the role of pyrin and TecA in pathogenesis. Xu et al. found by histopathology that an increase in inflammation seen in C57BL/6 mice infected with BcJ2315 was not observed in isogenic *Mefv*^−/−^ mice, suggesting that early immune cell influx to the lungs, and subsequent immunopathology, was pyrin dependent (22). Results of a similar infection procedure showed that TecA catalytic activity was required for BcJ2315 to cause immune cell recruitment and injury to the lungs of C57BL/6 mice (26). Although these results suggest that TecA acts as a virulence factor by triggering assembly of the pyrin inflammasome, leading to inflammatory cell recruitment and lung immunopathology, other measurements of disease (e.g., pathogen burdens and lethality) have not been examined to confirm this concept. Additionally, the roles of pyrin and TecA in B. cenocepacia pathogenesis have only been investigated with BcJ2315 and have not been studied in mice lacking Cftr function, which are known to have increased susceptibility to lung infections (14, 27, 28). Here, we used B. cenocepacia strain AU1054 (BcAU1054), isolated from the bloodstream of a CF patient in the United States (15), to study pyrin and TecA and their virulence roles during lung infection of C57BL/6 mice and mice with the F508del mutation in *Cftr*.

## RESULTS

### TecA is a virulence factor during BcAU1054 lung infection in WT mice.

To study B. cenocepacia pathogenesis and immunity, we established a mouse lung infection model with BcAU1054 (Table 1), a genomovar IIIB, PHDC lineage clinical isolate (15). In initial experiments, C57BL/6 (wild-type [WT]) mice were mock infected or infected with 5 × 10^7^ CFU of BcAU1054 by oropharyngeal (o.p.) aspiration, and at 12 h postinfection immunohistochemistry (IHC) was performed on lung sections using mouse antisera to B. cenocepacia. As shown in Fig. S1 in the supplemental material, B. cenocepacia antiserum staining was only detected in infected sections and was concentrated in what appeared to be phagocytes primarily located in the alveoli. This IHC staining pattern for B. cenocepacia in phagocytes is similar to what has been reported for infected human and mouse lung sections (12, 13).

**TABLE 1:** Bacterial strains used in this study

Strain name (abbreviated name)	Source or reference
Burkholderia cenocepacia AU1054 (BcAU1054)	15
Burkholderia cenocepacia AU1054 Δ*hcp* (Δ*hcp*)	15
Burkholderia cenocepacia AU1054 Δ*tecA* (Δ*tecA*)	This study
Burkholderia cenocepacia AU1054 Δ*tecA*::*tecA_WT_* (Δ*tecA*::*tecA*)	This study
Burkholderia cenocepacia AU1054 Δ*tecA*::*tecA_C41A_* (Δ*tecA*::*tecA_C41A_*)	This study
Burkholderia cenocepacia J2315 (BcJ2315)	George O’Toole

10.1128/mBio.02098-21.1 FIG S1 IHC staining of WT mouse lung sections infected with BcAU1054. IHC was performed with control serum (A) or BcAU1054 antisera (B) on fixed right lung lobe sections from a WT mouse left uninfected (mock) or infected by o.p. instillation with 5 × 10^7^ CFU of BcAU1054 for 12 h. Representative images captured by light microscopy are shown at ×10 or ×40 magnification. Download FIG S1, PDF file, 0.3 MB. Copyright © 2021 Loeven et al. 2021 Loeven et al. https://creativecommons.org/licenses/by/4.0/ This content is distributed under the terms of the Creative Commons Attribution 4.0 International license.

**FIG 1:**
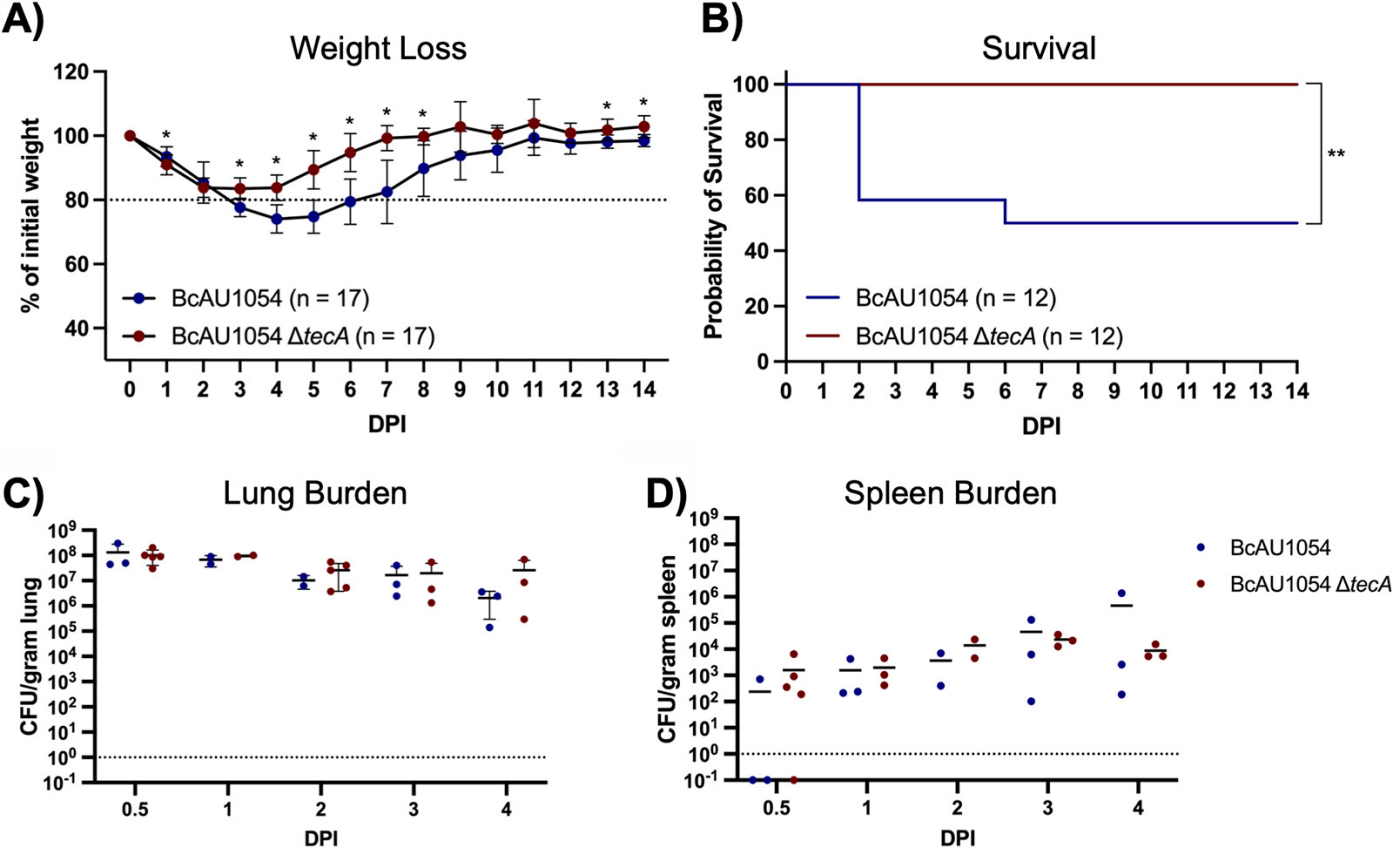
TecA enhances weight loss and lethality in WT mice infected with BcAU1054. (A) Weight loss of WT mice infected by o.p. instillation with 5 × 10^7^ CFU of BcAU1054 or Δ*tecA* mutant. Mice were weighed on D_0_ and daily for 14 dpi. Results were pooled from 3 independent experiments. *n*, total mice infected. Data are represented as percentages of initial weight. Error bars are standard deviations. Days on which the two groups differed significantly (*P* <  0.05, Welch’s *t* test) are marked with an asterisk. (B) Percent survival monitored for 14 dpi of WT mice infected with BcAU1054 or Δ*tecA* mutant. Results were pooled from 3 independent experiments. *n*, total mice infected. **, *P* = 0.0054 by log-rank (Mantel-Cox) test. (C and D) Lung (C) and spleen (D) burdens at the indicated dpi in WT mice infected with BcAU1054 or Δ*tecA* mutant. Results are pooled from multiple experiments, and each point represents a value obtained from an individual mouse and data are represented as number of CFU per gram of tissue. Lines are the medians.

To study the role of TecA in pathogenesis, *tecA* was deleted from BcAU1054 (Δ*tecA* mutant) (Table 1). Groups of WT mice were then infected with BcAU1054 or the Δ*tecA* mutant, and weight and survival were monitored for 14 days. Compared to mice infected with the Δ*tecA* mutant, B. cenocepacia AU1054-infected mice experienced significant weight loss (Fig. 1A). The weight loss data in Fig. 1A were used to estimate the average change in weight from the previous day (log_2_), and results were displayed by locally weighted scatterplot smoothing (LOESS) (data not shown). Using the asymptotic Wilcoxon-Mann-Whitney test, it was determined that the mice infected with the Δ*tecA* mutant regained weight significantly earlier than those infected with BcAU1054 (*P*  = 0.007201). Fifty percent of the mice infected with BcAU1054 succumbed to the disease, while the Δ*tecA* mutant was significantly less virulent (Fig. 1B), indicating that TecA is a virulence factor. In different sets of experiments, the percentages of WT mice that died from BcAU1054 infection ranged from 50% (Fig. 1B) to 20% (Fig. S4B), while the Δ*tecA* mutant was reproducibly avirulent. To demonstrate that the avirulent phenotype of the Δ*tecA* mutant was due to loss of TecA enzymatic activity, we complemented the Δ*tecA* mutant with wild-type *tecA* (Δ*tecA*::*tecA_WT_*) or catalytically inactive *tecA* (Δ*tecA*::*tecA_C41A_*) (Table 1) and carried out mouse infections with the resulting strains. The avirulent phenotype of the Δ*tecA* mutant was complemented by *tecA*_WT_ but not *tecA_C41A_*, as determined by the weight loss assay (Fig. S2), demonstrating that the enzymatic activity of TecA is required for virulence. To examine how TecA promotes virulence, CFU assays were performed on lungs and spleens of mice infected with BcAU1054 or the Δ*tecA* mutant over a time course. There was no trend indicating a difference in the numbers of BcAU1054 and Δ*tecA* mutant CFU detected over the time course up to day 4 (Fig. 1C and D), suggesting that TecA does not increase the burden of B. cenocepacia in the lung or dissemination to the spleen.

10.1128/mBio.02098-21.2 FIG S2 Enzymatic activity of TecA is necessary for exacerbated weight loss in WT mice. WT mice were infected o.p. with 5 × 10^7^ CFU of BcAU1054, Δ*tecA*, Δ*tecA*::*tecA_WT_*, or Δ*tecA*::*tecA_C41A_* mutant and weighed on D_0_ and daily for 14 dpi. Data are represented as percentage of initial weight. Error bars are standard deviations. Days on which mice infected with Δ*tecA*::*tecA_WT_* or Δ*tecA*::*tecA_C41A_* mutant differed significantly (*n* = 6, *P* < 0.05, Welch’s *t* test) are marked with an asterisk. Results are from one experiment in which none of the mice died. *n*, total mice infected. Download FIG S2, PDF file, 0.2 MB. Copyright © 2021 Loeven et al. 2021 Loeven et al. https://creativecommons.org/licenses/by/4.0/ This content is distributed under the terms of the Creative Commons Attribution 4.0 International license.

10.1128/mBio.02098-21.4 FIG S4 Pyrin is dispensable for BcAU1054 virulence in WT mice. (A) Weight loss of WT and *Mefv*^−/−^ mice infected o.p. with 5 × 10^7^ CFU of BcAU1054. Mice were weighed on D_0_ and daily for 14 dpi. Results were pooled from 2 independent experiments. *n*, total mice infected. Data are represented as a percentage of initial weight. Error bars are standard deviations. (B) Percent survival monitored for 14 dpi of WT and *Mefv*^−/−^ mice infected with BcAU1054. Results were pooled from 2 independent experiments. *n*, total mice infected. ns, not significant by log-rank (Mantel-Cox) test. (C and D) Lung and spleen burden at the indicated day postinfection in WT and *Mefv*^−/−^ mice infected with BcAU1054. Results are from one experiment, and each point represents a value obtained from an individual mouse. Data are represented as number of CFU per gram of tissue. Lines are the medians. ns, not significant by Mann-Whitney test. The mice used in these experiments were not controlled using littermates or cohousing. Download FIG S4, PDF file, 0.3 MB. Copyright © 2021 Loeven et al. 2021 Loeven et al. https://creativecommons.org/licenses/by/4.0/ This content is distributed under the terms of the Creative Commons Attribution 4.0 International license.

**FIG 2:**
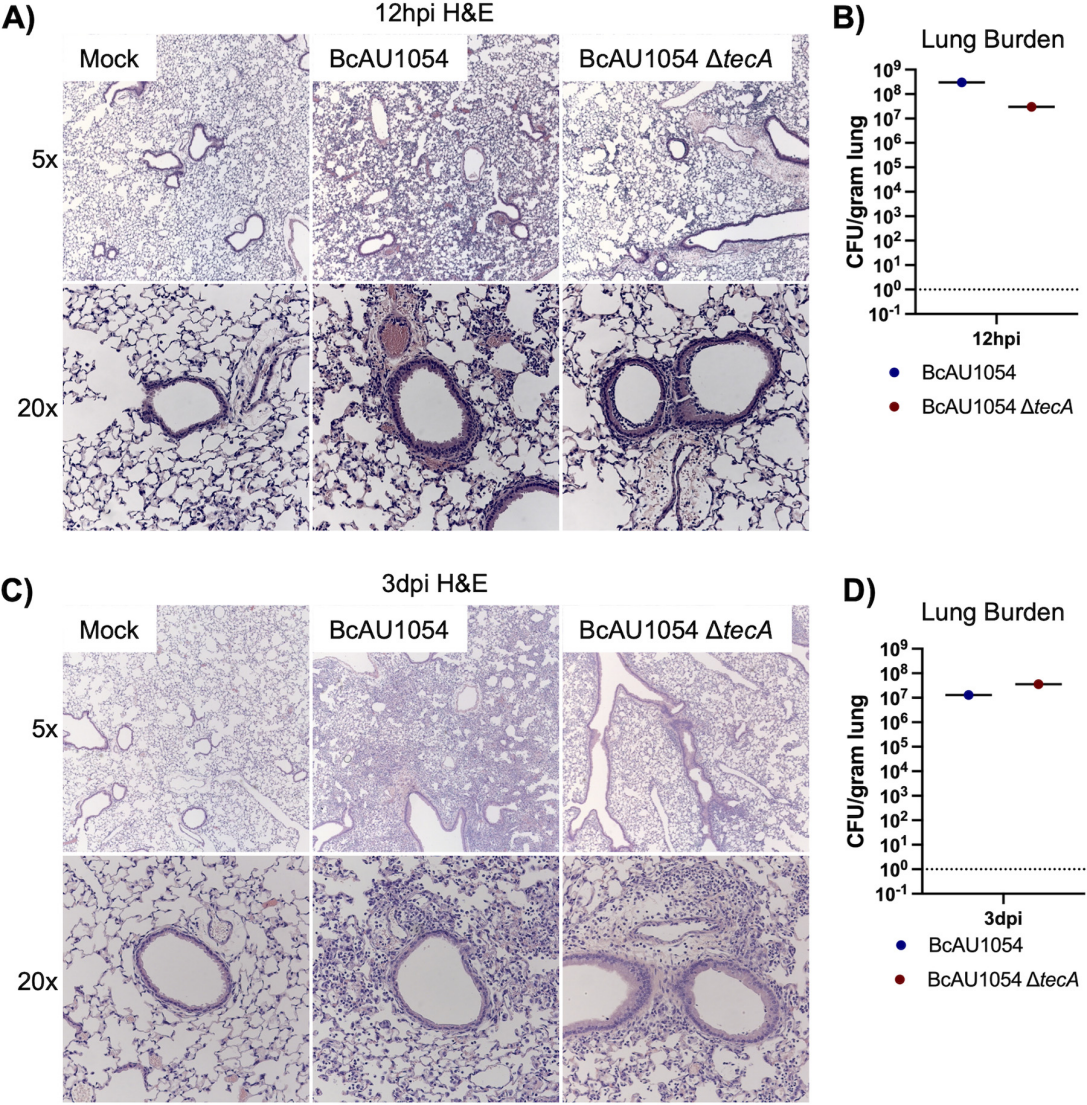
Lung inflammation in WT mice infected with BcAU1054 or Δ*tecA* mutant. (A and C) Representative H&E images of the four right lung lobe sections from a WT mouse left uninfected (mock) or infected o.p. with 5 × 10^7^ CFU of BcAU1054 or Δ*tecA* mutant at 12 hpi (A) and 3 dpi (C). Light microscopy images are shown at ×5 and ×20 magnification. Burdens of BcAU1054 or Δ*tecA* mutant in the infected left lungs of the mice analyzed in panels A and C are shown in panels B and D, respectively. Data are represented as number of CFU per gram of lung.

**FIG 3:**
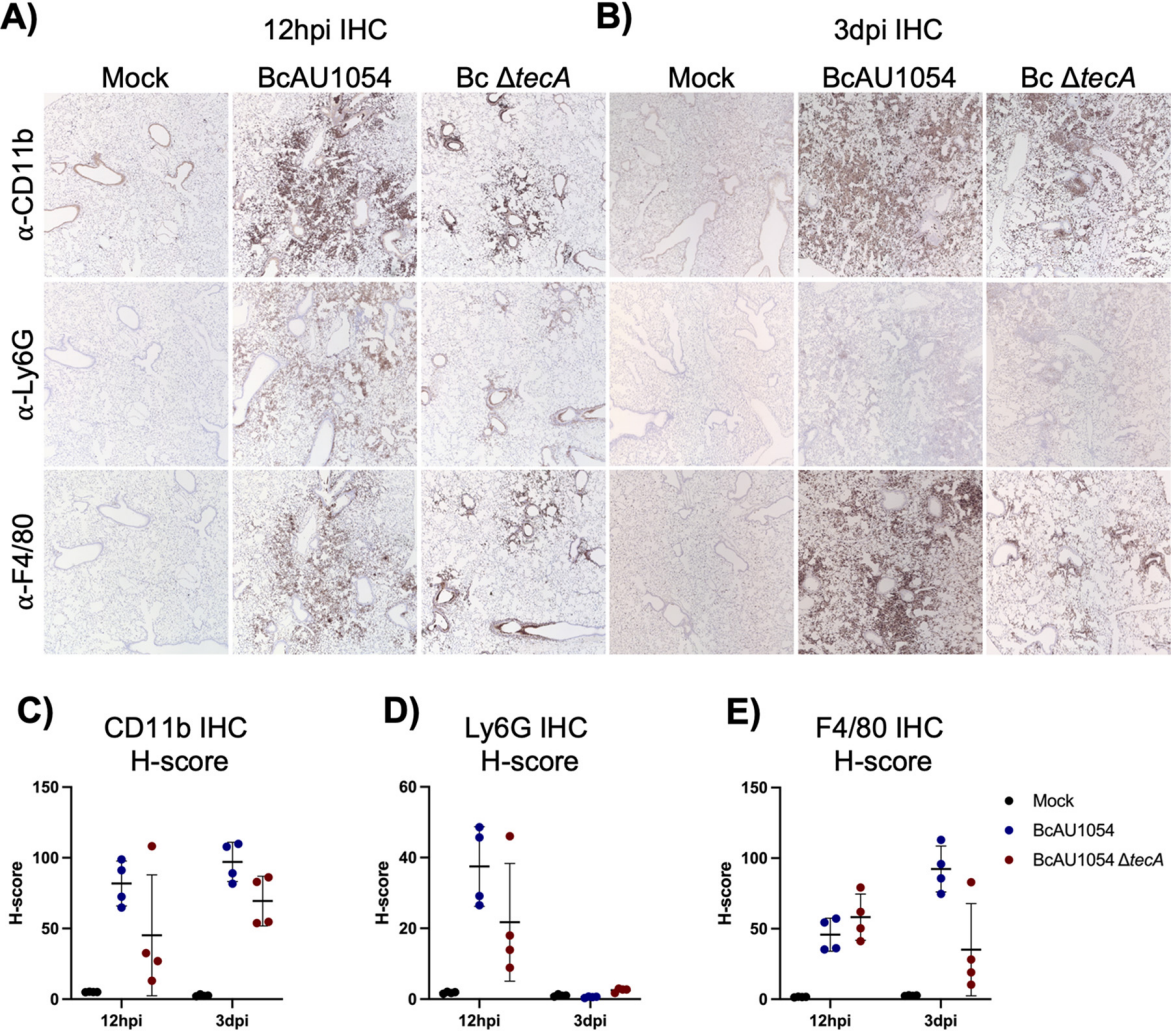
TecA enhances recruitment of neutrophils and inflammatory monocyte-derived macrophages to lungs in WT mice infected with BcAU1054. IHC was performed on sections from the right lung lobes analyzed in Fig. 2 using CD11b, Ly6G, and F4/80 antibodies as indicated. Representative IHC images captured by light microscopy at ×5 magnification are shown. Quantitative IHC image analysis results normalized using H-scores are shown for CD11b (C), for Ly6G (D), and for F4/80 (E). Each data point represents the H-score for each of the four different lobe sections. Error bars are standard deviations, and lines are means.

Staining of lung sections with hematoxylin and eosin (H&E) showed mild edema and peribronchiolar and alveolar inflammatory infiltrates that appeared more extensive in the BcAU1054 infections than the Δ*tecA* mutant, which had regions that appeared essentially uninvolved, at both 12 h (Fig. 2A and B) and 3 days (Fig. 2C and D) postchallenge. These results are in line with those of Aubert et al., who used a similar infection model with BcJ2315 in WT mice and showed that TecA increases infiltration of inflammatory cells and lung damage at 12 h (26). Semiquantitative scoring of blinding images by a pathologist indicated that infection by both BcAU1054 and the Δ*tecA* mutant caused similar neutrophilic and mononuclear cell infiltration and damage in the bronchioles and alveoli (Table S1). To better quantify the cell infiltration, IHC analysis of lung sections using antibodies to CD11b, Ly6G, and F4/80 was carried out. Representative images are shown in Fig. 3A and B, and quantitative analysis results normalized using H-scores are shown in Fig. 3C to E. Results showed that BcAU1054 TecA enhanced neutrophil (CD11b^+^ Ly6G^+^) infiltration at 12 h postinfection (Fig. 3A, C, and D) and inflammatory monocyte-derived macrophage (CD11b^+^ F4/80^+^) infiltration at 3 days postinfection (dpi) (Fig. 3B, C, and E). By 3 days postinfection, the neutrophil numbers in the lungs had decreased to background levels under both infection conditions (Fig. 3B and D), while numbers of inflammatory monocyte-derived macrophages had increased at this time point in response to infection with BcAU1054 (Fig. 3B, C, and E). These results are consistent with BcAU1054 TecA exacerbating an acute lethal bronchopneumonia that is dominated by a sustained increase in F4/80^+^ inflammatory monocyte-derived macrophages at day 3 postinfection.

10.1128/mBio.02098-21.7 TABLE S1 Summary of semi-quantitative scoring of lung inflammation and damage. Download TABLE S1, PDF file, 0.1 MB. Copyright © 2021 Loeven et al. 2021 Loeven et al. https://creativecommons.org/licenses/by/4.0/ This content is distributed under the terms of the Creative Commons Attribution 4.0 International license.

### TecA is a virulence factor during BcAU1054 lung infection in CF mice.

**FIG 4:**
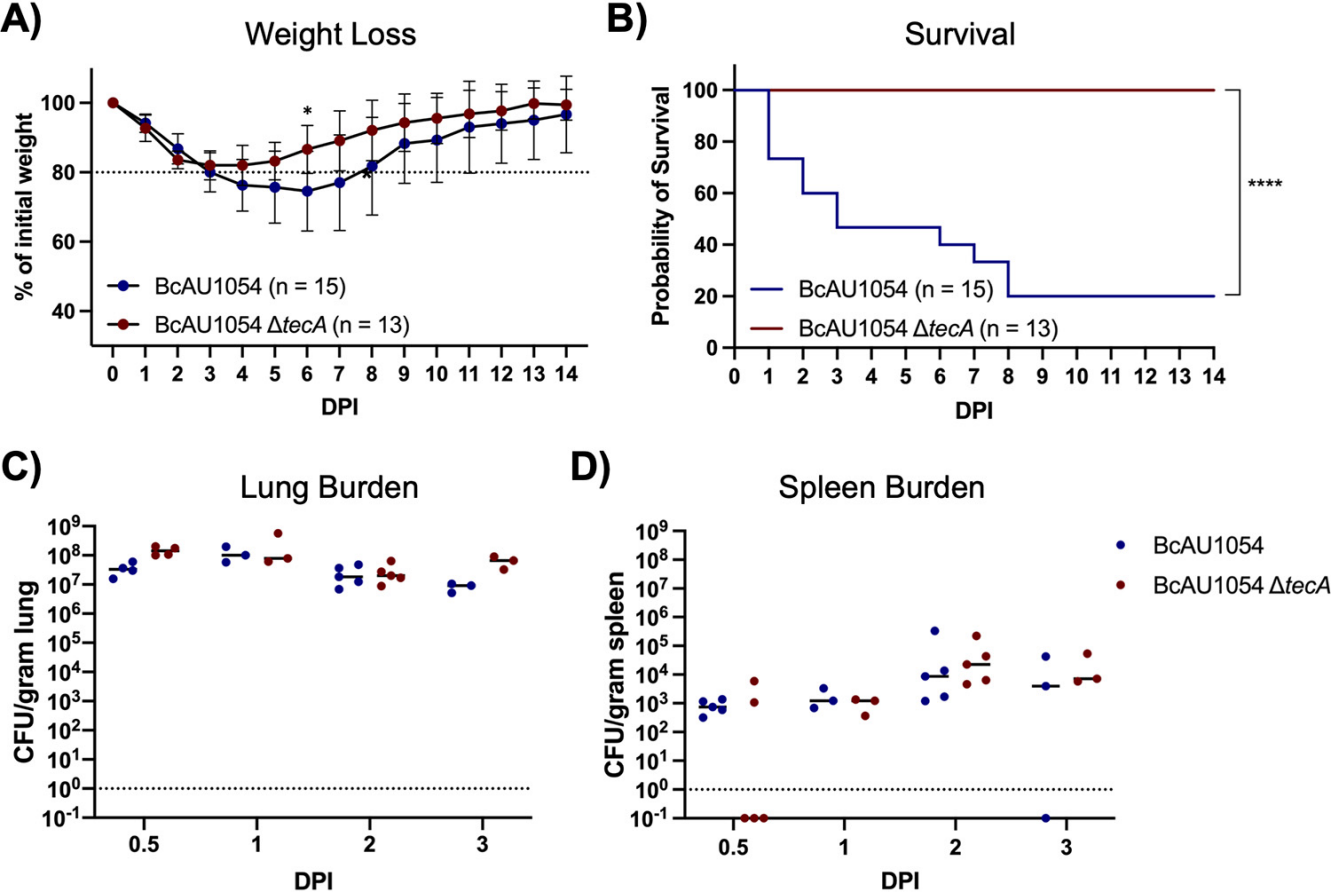
TecA enhances weight loss and lethality in *Cftr^F508del^* mice infected with BcAU1054. (A) Weight loss of *Cftr^F508del^* mice infected o.p. with 5 × 10^7^ CFU of BcAU1054 or Δ*tecA* mutant. Mice were weighed on D_0_ and daily for 14 dpi. Results were pooled from 5 independent experiments. *n*, total mice infected. Data are represented as percentages of initial weight. Error bars are standard deviations. Days on which the two groups differed significantly (*P* <  0.05, Welch’s *t* test) are marked with an asterisk. (B) Percent survival monitored for 14 dpi of *Cftr^F508del^* mice infected with BcAU1054 or Δ*tecA* mutant. Results were pooled from five independent experiments. *n*, total mice infected. ****, *P* < 0.0001 by log-rank (Mantel-Cox) test. (C and D) Lung (C) and spleen (D) burden at the indicated dpi in *Cftr^F508del^* mice infected with BcAU1054 or Δ*tecA* mutant. Results were pooled from multiple experiments, and each point represents a value obtained from an individual mouse. Data are represented as number of CFU per gram of tissue. Lines are the medians.

**FIG 5:**
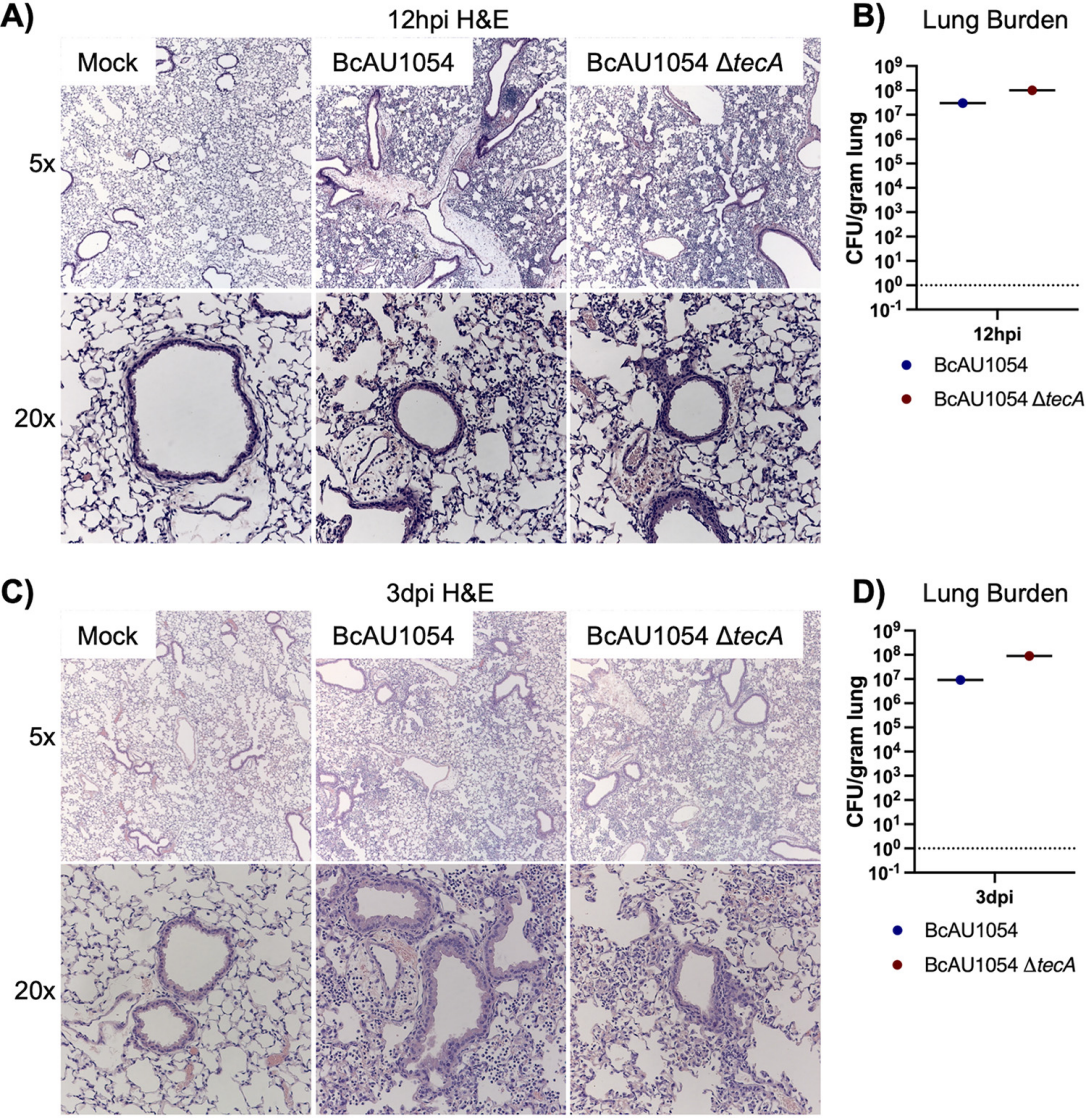
Lung inflammation in *Cftr^F508del^* mice infected with BcAU1054 or Δ*tecA* mutant. (A) Representative H&E images of the four right lung lobe sections from a *Cftr^F508del^* mouse left uninfected (mock) or infected o.p. with 5 × 10^7^ CFU of BcAU1054 or Δ*tecA* mutant at 12 hpi (A) or 3 dpi (C). Light microscopy images are shown at ×5 and ×20 magnification. Burdens of BcAU1054 or Δ*tecA* mutant in the infected left lungs of the mice analyzed in panels A and C are shown in panels B and D, respectively. Data are represented as number of CFU per gram of lung.

**FIG 6:**
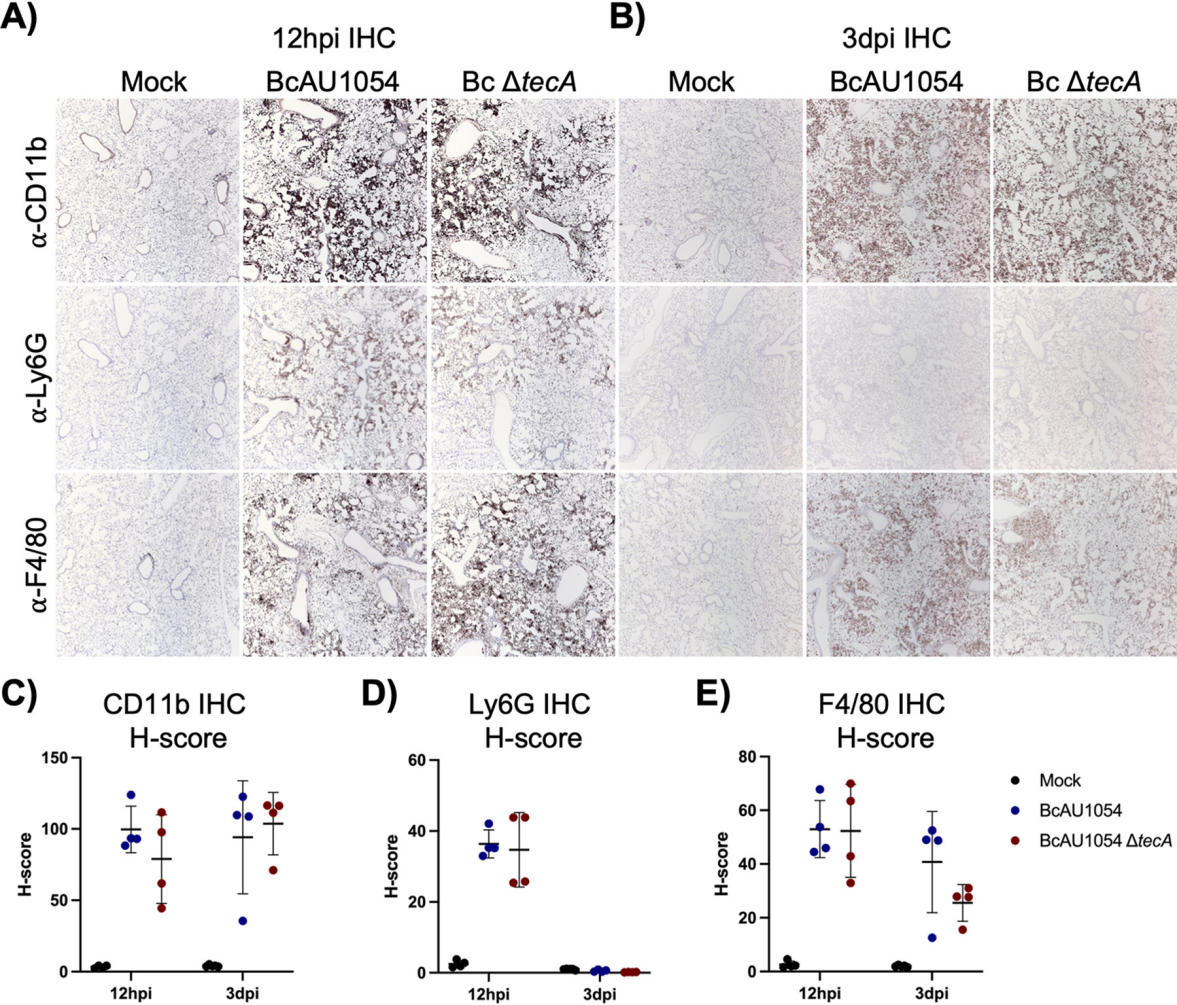
TecA enhances recruitment of inflammatory monocyte-derived macrophages to lungs in *Cftr^F508del^* mice infected with BcAU1054. IHC was performed on sections from the right lung lobes analyzed in Fig. 5 using CD11b, Ly6G, and F4/80 antibodies as indicated. Representative IHC images captured by light microscopy at ×5 magnification are shown. Quantitative IHC image analysis results normalized using H-scores are shown for CD11b (C), Ly6G (D), and F4/80 (E). Each data point represents the H-score for each of the four different lobe sections. Error bars are standard deviations, and lines are means.

**FIG 7:**
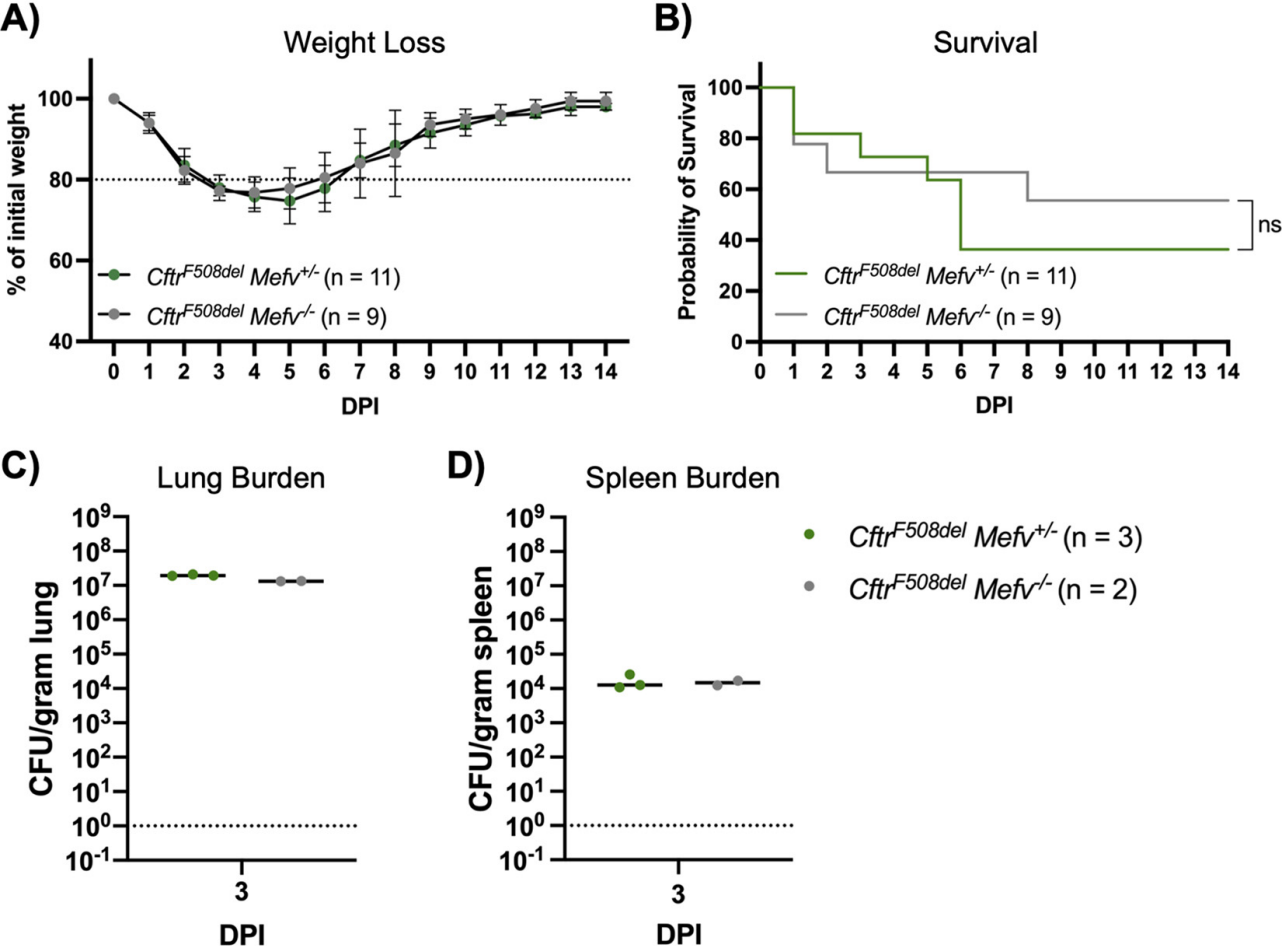
Pyrin is dispensable for BcAU1054 virulence in *Cftr^F508del^* mice. (A) Weight loss of *Cftr^F508del^ Mefv*^+/^*^−^* and *Cftr^F508del^ Mefv*^−/−^ mice infected o.p. with 5 × 10^7^ CFU of BcAU1054. Mice were weighed on D_0_ and daily for 14 dpi. Results were pooled from 3 independent experiments. *n*, total mice infected. Data are represented as percent initial weight. Error bars are standard deviations. (B) Percent survival monitored for 14 days postinfection of *Cftr^F508del^ Mefv^+/−^* and *Cftr^F508del^ Mefv*^−/−^ mice infected with BcAU1054. Results were pooled from three independent experiments. *n*, total mice infected. ns, not significant by log-rank (Mantel-Cox) test. (C and D) Lung (C) and spleen (D) burden at 3 dpi in *Cftr^F508del^ Mefv^+/−^* and *Cftr^F508del^ Mefv*^−/−^ mice infected with BcAU1054. Data are represented as number of CFU per gram of tissue. Lines are the medians.

Previous studies have indicated that mice lacking Cftr function have increased susceptibility to B. cenocepacia lung infections (14, 27, 28). To examine this possibility in our infection model with BcAU1054, *Cftr^F508del^* (*Cftr^em1Cwr^*) mice were obtained from the Case Western Reserve University CF Mouse Model Core. *Cftr^F508del^* mice were infected with BcAU1054 or the Δ*tecA* mutant as described above. *Cftr^F508del^* mice infected with BcAU1054 had increased weight loss compared to the Δ*tecA* mutant (Fig. 4A), although this difference was not significant when the data were displayed by LOESS and analyzed using the asymptotic Wilcoxon-Mann-Whitney test (data not shown). TecA was required for lethality (Fig. 4B) without impacting lung or spleen CFU numbers over the first 3 days of infection (Fig. 4C and D). In different sets of experiments, the percentages of *Cftr^F508del^* mice that died from BcAU1054 infection ranged from 80% (Fig. 4B) to ∼50% (see Fig. 7B), suggesting that the *Cftr^F508del^* mice are more susceptible to lethal disease than WT mice. BcAU1054 and Δ*tecA* mutant infection increased inflammation in the lungs of *Cftr^F508del^* mice at 12 h and 3 days postinfection, and the H&E staining results were similar to those obtained in WT mice (compare Fig. 5 with Fig. 2). Lung sections from 12 h postinfection and 3 days postinfection were also stained for IHC using antibodies to CD11b, Ly6G, or F4/80 (Fig. 6). Overall, the staining pattern was similar to that in WT mice, although there was no trend toward increased Ly6G^+^ neutrophils in the BcAU1054- compared to Δ*tecA*-infected lung sections at 12 h (compare Fig. 6D with Fig. 3D). In summary, BcAU1054 TecA functioned as a virulence factor during lung infection in *Cftr^F508del^* mice.

### Pyrin is dispensable for B. cenocepacia virulence during lung infection in mice.

Having established that TecA is a virulence factor, experiments were carried out to determine the importance of the pyrin inflammasome for pathogenesis during BcAU1054 lung infection in mice. To confirm that TecA in BcAU1054 can trigger pyrin inflammasome assembly in WT and *Cftr^F508del^* backgrounds, *in vitro* infection experiments were carried out using lipopolysaccharide (LPS)-primed bone marrow-derived macrophages (BMDMs). WT and *Cftr^F508del^* BMDMs were left uninfected or infected at a multiplicity of infection (MOI) of 20 for 90 min with BcAU1054 or Δ*tecA*, Δ*tecA*::*tecA_WT_*, or Δ*tecA*::*tecA_C41A_* mutant. Purified TcdB from Clostridium difficile was used as a positive control for pyrin inflammasome activation. Results of Western blotting and enzyme-linked immunosorbent assay (ELISA) were used to monitor the outcomes of the infections or intoxications. In its inactive conformation, murine pyrin is phosphorylated at serine 205 (p-S205) and serine 241 (29). Dephosphorylation of these sites appears to trigger activation of pyrin (29, 30). The phosphorylation status of pyrin was determined by Western blotting BMDM lysates for total pyrin and p-S205 pyrin (30). WT and *Cftr^F508del^* BMDMs infected with strain BcAU1054 or Δ*tecA*::*tecA_WT_* mutant or intoxicated with TcdB had decreased levels of p-S205 pyrin, whereas those infected with the Δ*tecA* or Δ*tecA*::*tecA_C41A_* mutant retained p-S205 (Fig. S3A). Pro-IL-1β was detected by Western blotting at similar levels in LPS-primed BMDMs under all conditions (Fig. S3A). ELISA results confirmed that the pyrin inflammasome was assembled in response to TecA or TcdB activity in WT or *Cftr^F508del^* BMDMs, resulting in processing and release of IL-1β by both cell types (Fig. S3B and D).

10.1128/mBio.02098-21.3 FIG S3 B. cenocepacia TecA activates the pyrin inflammasome in WT and *Cftr^F508del^* BMDMs. (A) Whole-cell lysates from WT and *Cftr^F508del^* BMDMs left unprimed or primed with LPS and left uninfected or infected at an MOI of 20 with BcAU1054, Δ*tecA*, Δ*tecA*::*tecA_WT_*, or Δ*tecA*::*tecA_C41A_* mutant or intoxicated with TcdB for 90 min were analyzed by Western blotting with antibodies to total pyrin, p-S205 pyrin, pro-IL-1β, or β-actin (loading control). (B) IL-1β quantified by ELISA in cell supernatants collected from WT and *Cftr^F508del^* BMDMs left unprimed or primed with LPS and left uninfected or infected with B. cenocepacia or intoxicated with TcdB as for panel A. Data shown are from at least three independent experiments. Differences compared to BcAU1054 were not significant as determined by two-way ANOVA (Tukey’s multiple-comparison test) (data not shown) analysis on both BMDM genotype groups. Differences compared to BcAU1054 were significant in WT but not *Cftr^F508del^* as determined by one-way ANOVA (Tukey’s multiple-comparison test) analysis on the individual BMDM genotype groups. (C) Whole-cell lysates from *Cftr^F508del^ Mefv^+/+^*, *Cftr^F508del^ Mefv^+/−^*, and *Cftr^F508del^ Mefv*^−/−^ BMDMs primed with LPS were analyzed by Western blotting with antibodies to total pyrin and β-actin (loading control). (D) IL-1β quantified by ELISA in cell supernatants collected from *Cftr^F508del^ Mefv^+/+^*, *Cftr^F508del^ Mefv^+/−^*, and *Cftr^F508del^ Mefv*^−/−^ BMDMs primed with LPS and left uninfected or infected with BcAU1054, Δ*tecA* mutant, or Δ*hcp* mutant or intoxicated with TcdB for 90 min. Data shown are from at least three independent experiments. Significance determined by two-way ANOVA (Tukey’s multiple-comparison test) comparing to BcAU1054 within BMDM genotypes and between BMDM genotypes as indicated by brackets. (E) IL-1β quantified by ELISA in cell supernatant collected from WT and *Mefv*^−/−^ BMDMs primed with LPS and left uninfected or infected with BcJ2315 or intoxicated with TcdB for 90 min. Data shown are from at least six (WT) or two (*Mefv*^−/−^) independent experiments. Significance comparing BcJ2315 to uninfected determined by Mann-Whitney. In panels B, D, and E, error bars represent standard deviations. ****, *P* < 0.0001; ***, *P* < 0.001; *, *P* < 0.05; ns, nonsignificant. Download FIG S3, PDF file, 1.0 MB. Copyright © 2021 Loeven et al. 2021 Loeven et al. https://creativecommons.org/licenses/by/4.0/ This content is distributed under the terms of the Creative Commons Attribution 4.0 International license.

The *Cftr^F508del^* mice were crossed to *Mefv*^−/−^ mice, and the offspring were infected with BcAU1054 to investigate the role of the pyrin inflammasome for pathogenesis during BcAU1054 lung infection. As shown in Fig. 7, there was no difference in weight loss (A), survival (B), or lung (C) or spleen (D) CFU burdens (day 3) when *Cftr^F508del^ Mef*^+/^*^−^* or *Cftr^F508del^ Mefv*^−/−^ mice were infected with BcAU1054. To determine if *Mef*^+/^*^−^* mice produce pyrin and secrete IL-1β to an extent similar to that of the *Mefv^+/+^* background, *Cftr^F508del^ Mefv^+/+^*, *Cftr^F508del^ Mef*^+/^*^−^*, and *Cftr^F508del^ Mefv*^−/−^ BMDMs were prepared and LPS primed. Western blotting of uninfected BMDM lysates showed that total pyrin was produced in *Cftr^F508del^ Mefv^+/−^* BMDMs at the expected ∼2-fold smaller amounts than *Cftr^F508del^ Mefv^+/+^* BMDMs (Fig. S3C). The BMDMs were then infected as described above with BcAU1054, Δ*tecA*, or the Δ*hcp* T6SS-1-deficient mutant (Table 1) or intoxicated with TcdB. ELISA results showed that *Cftr^F508del^ Mefv^+/−^* BMDMs released ∼2-fold smaller amounts of IL-1β in response to TecA compared to *Cftr^F508del^ Mefv^+/+^* BMDMs (Fig. S3D).

To extend these results to the non-CF background, WT or *Mefv*^−/−^ mice were infected with BcAU1054. As shown in Fig. S4, there was no difference in weight loss (Fig. S4A), survival (Fig. S4B), or organ CFU burdens at day 3 (Fig. S4C and D) when WT or *Mefv*^−/−^ mice were infected with BcAU1054. Lung sections from these mice infected with BcAU1054 were examined after H&E staining, and no difference in inflammatory cell infiltration was evident between WT and *Mefv*^−/−^ genotypes (Fig. S5). Production of pyrin in inflammatory cells obtained by bronchoalveolar lavage (BAL) of WT mice infected with BcAU1054 or the Δ*tecA* mutant was confirmed by immunoblotting (Fig. S6). Thus, our results with BcAU1054 (genomovar IIIB, PHDC lineage) differed from Xu et al., who reported that inflammatory cell infiltration into lungs of mice intranasally infected with BcJ2315 (genomovar IIIA, ET12 lineage) required the pyrin inflammasome (22).

10.1128/mBio.02098-21.5 FIG S5 Pyrin is dispensable for lung inflammation in WT mice infected with BcAU1054. (A) Representative H&E images of the four right lung lobe sections from a WT or *Mefv*^−/−^ mouse uninfected (mock) or infected o.p. with 5 × 10^7^ CFU BcAU1054 at 3 dpi. Light microscopy images are shown at ×5 and ×20 magnification. (B) Lung burdens of BcAU1054 in infected left lungs of mice analyzed in panel A. Data are represented as number of CFU per gram of lung. Download FIG S5, PDF file, 0.5 MB. Copyright © 2021 Loeven et al. 2021 Loeven et al. https://creativecommons.org/licenses/by/4.0/ This content is distributed under the terms of the Creative Commons Attribution 4.0 International license.

10.1128/mBio.02098-21.6 FIG S6 Pyrin protein levels in BAL cell lysates from infected WT mice. WT mice were left uninfected (mock) or were infected o.p. with 5 × 10^7^ CFU of BcAU1054 or Δ*tecA* mutant, and BALF was collected at indicated time points. Lysates from BAL cells were normalized for total protein and immunoblotted for total pyrin or β-actin (loading control). Download FIG S6, PDF file, 0.3 MB. Copyright © 2021 Loeven et al. 2021 Loeven et al. https://creativecommons.org/licenses/by/4.0/ This content is distributed under the terms of the Creative Commons Attribution 4.0 International license.

To determine if the discrepancy between our results and those of Xu et al. was due to B. cenocepacia strain differences, *Mefv^+/−^* and *Mefv*^−/−^ mice were infected o.p. with 5 × 10^7^ CFU of BcJ2315 (Table 1) (22). BcJ2315 appeared to be less virulent than BcAU1054, as the weight loss was less severe in the infected mice (Fig. S7A). Nevertheless, there was no difference in weight loss or survival for *Mefv^+/−^* and *Mefv*^−/−^ mice infected with BcJ2315 (Fig. S7A and B). In addition, H&E-stained lung sections from *Mefv^+−^* and *Mefv*^−/−^ mice showed no difference in inflammatory cell infiltration at 12 h and 3 days postinfection with BcJ2315 (Fig. S8A and C). We confirmed by IL-1β ELISA that our isolate of BcJ2315 triggered assembly of the pyrin inflammasome in LPS-primed BMDMs infected *in vitro* (Fig. S3E). Thus, the requirement for pyrin in lung inflammation previously reported with the BcJ2315 strain (22) was not reproduced in our mouse infection model.

10.1128/mBio.02098-21.7 FIG S7 Pyrin is dispensable for weight loss in WT mice infected with BcJ2315. (A) Weight loss of *Mefv^+/−^* and *Mefv*^−/−^ mice infected o.p. with 5 × 10^7^ CFU BcJ2315. Mice were weighed on D_0_ and daily for 14 dpi. Data are represented as percent initial weight. Error bars are standard deviations. (B) Percent survival monitored for 14 dpi of *Mefv^+/−^* and *Mefv*^−/−^ mice infected with BcJ2315. Results shown are from one experiment. *n*, total mice infected for both panels A and B. Download FIG S7, PDF file, 0.2 MB. Copyright © 2021 Loeven et al. 2021 Loeven et al. https://creativecommons.org/licenses/by/4.0/ This content is distributed under the terms of the Creative Commons Attribution 4.0 International license.

10.1128/mBio.02098-21.8 FIG S8 Pyrin is dispensable for lung inflammation in WT mice infected with BcJ2315. Representative H&E images of the four right lung lobe sections from a *Mefv^+/−^* or *Mefv*^−/−^ mouse left uninfected (mock) or infected with 5 × 10^7^ CFU BcJ2315 for 12 h (A) or 3 days (C). Light microscopy images are shown at ×5 and ×20 magnification. Burdens of BcJ2315 in the infected left lungs analyzed in panels A and C are shown in panels B and D, respectively. Data are represented as number of CFU per gram of lung. Download FIG S8, PDF file, 0.8 MB. Copyright © 2021 Loeven et al. 2021 Loeven et al. https://creativecommons.org/licenses/by/4.0/ This content is distributed under the terms of the Creative Commons Attribution 4.0 International license.

## DISCUSSION

The underlying basis for the pathogenesis of B. cenocepacia during lung infection remains to be fully understood. Aubert et al. demonstrated that TecA is critical for BcJ2315 to cause inflammatory cell recruitment and damage to lungs in WT mice (26). We have extended these results in the following ways. (i) We show that TecA is a virulence factor that exacerbates weight loss and lethality in addition to inflammatory cell recruitment to lungs. (ii) Our results were obtained with BcAU1054, a genomovar IIIB, PHDC lineage strain distinct from the BcJ2315 genomovar IIIA, ET12 lineage strain, suggesting that TecA is a virulence factor in all members of this species. (iii) We demonstrate that TecA is a virulence factor in *Cftr*^*F508del*^ mice, which provide a useful CF animal model. (iv) Our findings suggest that the presence of TecA in B. cenocepacia is one reason why this species is most commonly responsible for cepacia syndrome (1). (v) Our data support the idea that TecA is the founding member of a family of virulence factors in opportunistic bacterial pathogens, based on the finding that other opportunistic bacterial pathogens encode functional TecA orthologs (26), including Chryseobacterium indologenes (31) and Ochrobactrum anthropi (32). Finally, our results are significant because there are only a few examples of T6SS effectors that have demonstrated anti-host virulence factor activity in a mammalian infection model (33).

In an unexpected difference from what has been published with BcJ2315 (22), we were not able to reproduce a requirement for pyrin in inflammatory cell recruitment to lungs during infection with either BcJ2315 or BcAU1054. Although BcJ2315 appears to be less virulent than BcAU1054, possibly due to the lack of O-antigen (26), this strain and BcAU1054 induced inflammatory cell recruitment to lungs independent of pyrin in our infection model. The TecA orthologs are 91% identical between the two strains (26), and both BcJ2315 and BcAU1054 trigger assembly of pyrin inflammasomes in infected BMDMs. Other experimental differences could be responsible for the discrepancy. While we used o.p. infections with 5 × 10^7^ CFU, Xu et al. used intranasal infections with the 2-fold higher dose of 1 × 10^8^ CFU of BcJ2315 (22). Another difference is that the *Mefv*^−/−^ mouse lines used in our study (34) and that of Xu et al. (22) are both on the C57BL/6 background but independently generated and housed in different animal facilities. It is conceivable that mouse microbiota differences associated with the different animal facilities used in our study and that of Xu et al. (22) are responsible for the discrepant results. It will be important to address this possibility in future studies.

Results of studies with BMDMs suggest that B. cenocepacia infection can trigger T6SS-1-dependent activation of the NLRP3 (17, 35) and noncanonical caspase-11 inflammasomes (36, 37) in addition to the pyrin inflammasome. We did not detect substantial amounts of IL-1β released from LPS-primed *Mefv*^−/−^ BMDMs infected with BcAU1054 or BcJ2315, indicating that pyrin-independent inflammasomes are not activated during these *in vitro* infections. In a recent study, C57BL/6 *Gsdmd*^−/−^ mice were infected o.p. with B. cenocepacia strain K65-2, and the authors reported no difference in survival compared to WT controls (36). These results are consistent with the idea that cleavage of GSDMD, which would occur downstream of NLRP3, caspase-11, or pyrin inflammasomes, is not essential for the virulence of B. cenocepacia during lung infection as measured by a survival assay. However, this study reported that after BcK56-2 infection, *Gsdmd*^−/−^ mice had lower lung inflammation by H&E staining than the WT at 48 h postchallenge (36), suggesting that an inflammasome is contributing to immunopathology under these conditions. Additionally, GSDMD has a major role in restricting replication of BcK56-2 in macrophages, and at 48 h postinfection *Gsdmd*^−/−^ mice had higher BcK56-2 organ burdens than the WT (36). Understanding how different inflammasome pathways contribute to pathogenesis or host protection during B. cenocepacia infections is an important goal in the field that will require additional research.

Although we demonstrated that pyrin is produced in BAL cells in response to BcAU1054 infection of WT mice, we have not attempted to determine if pyrin is activated by TecA in these cells. Aubert et al. obtained evidence that TecA triggers assembly of the pyrin inflammasome during systemic B. cenocepacia infection in mice. In an intraperitoneal infection model, a Δ*tecA* mutant exhibited increased spleen burdens (day 4 postchallenge) and lethality in WT mice compared to the parental BcJ2315 strain (26). However, in *Mefv*^−/−^ mice the Δ*tecA* mutant and parent exhibited similar lethality phenotypes, indicating that TecA triggers assembly of the pyrin inflammasome when infections are initiated systemically, although in this case it leads to a protective host response. It is possible that host phagocytes differentially control assembly of the inflammasome during B. cenocepacia infections in distinct organs, such that this pathway is activated by pyrin in spleen but not lung.

How TecA promotes virulence during B. cenocepacia lung infections remains to be determined. TecA does not increase BcAU1054 CFU in lungs, suggesting that it does not directly counteract bactericidal activities of phagocytes when the infection is initiated by this route. T6SS-1 does not reduce phagocytosis of B. cenocepacia, although it can inhibit uptake of secondary targets 60 min after infection (38), which is consistent with the idea that the bacteria exploit the macrophage intracellular environment as a replicative niche. There is evidence that B. cenocepacia can delay maturation of its phagosome in macrophages (39, 40), and recently this has been attributed to TecA (41), but it remains unclear if this activity is essential for survival of the bacteria in host phagocytes. There is also evidence that T6SS-1 inhibits assembly of NADPH oxidase complexes on B. cenocepacia-containing phagosomes in macrophages (35, 42). However, the fact that only patients with defects in NADPH oxidase activity, such as CF or chronic granulomatous disease, are highly susceptible to B. cenocepacia infections strongly argues that these bacteria do not efficiently inhibit superoxide production in phagocytes. In a direct comparison of BcK56-2 and a Burkholderia multivorans clinical isolate in stimulating the oxidative burst in human macrophages, both strains induced the oxidative burst at roughly equivalent magnitudes (43). Thus, BcK56-2, which encodes *tecA*, does not appear to inhibit the oxidative burst more efficiently than a *B. multivorans* strain lacking this effector (26). TecA also does not appear to increase dissemination of B. cenocepacia from the lung, based on spleen or liver CFU numbers of B. cenocepacia, although this result may be hard to interpret if increased dissemination is balanced against decreased bacterial survival due to protective pyrin inflammasome responses in spleen or liver.

It is possible that TecA primarily promotes virulence during B. cenocepacia infection by inducing lung immunopathology, as first observed by Aubert et al. (26). Immunopathology leading to lung failure could be the underlying basis for TecA-dependent weight loss and lethality in our infection model. A pyrin inflammasome-independent mechanism of immunopathology could be TecA-triggered cytokine or chemokine production, leading to increased numbers of F4/80^+^ inflammatory monocyte-derived macrophages in the B. cenocepacia-infected lung, as we observed by IHC. CCL2 is produced by a variety of cells and signals for the recruitment of Ccr2^+^ inflammatory monocytes out of the bone marrow. We hypothesize that CCL2-elicited Ccr2^+^ inflammatory monocytes play a role in TecA-induced immunopathology, as they may be the source of the F4/80^+^ cells in lung sections at 3 days postinfection with BcAU1054, and these cells have been demonstrated to be detrimental to the host in other lung infection contexts (44). Studies using mice in which Ccr2^+^ inflammatory monocytes are reduced or can be depleted are under way to test this hypothesis.

Bacterial toxins that inactivate Rho GTPases can trigger activation of mitogen-activated protein kinases (MAPKs) in phagocytes (45), representing an alternative mechanism by which TecA could induce immunopathology. It has been shown that TcdA and TcdB from *C. difficile* trigger activation of MAPK pathways in monocytes and intestinal epithelial cells, leading to release of IL-8 (45, 46). The activation of MAPK MK2 (pMK2) has been demonstrated in epithelial cells as well as in mouse and hamster intestines (46). IL-8 is a potent neutrophil chemoattractant, and secretion of IL-8 in the gut mucosa leads to injury (45, 46). Interestingly, IL-8 has also been shown to be secreted at higher levels in CF immune cells than non-CF cells and is present at higher levels in CF patient BAL samples, suggesting that there are alterations in MAPK signaling in CF (47, 48).

B. cenocepacia infection in *Cftr*^*F508del*^ mice does not recapitulate all aspects of the disease in CF patients (e.g., lack of preceding chronic infection and lung necrosis in the context of cepacia syndrome), but we consider it a reasonable and useful CF animal infection model. This model can be used to uncover the virulence mechanisms of TecA. In addition, this model can be used to better understand how immune deficiencies in CF contribute to susceptibility to B. cenocepacia lung infection. For example, does dysbiosis of the microbiota in CF contribute to risk for B. cenocepacia lung infection? Finally, this model can be used to understand mechanisms of protective immunity to B. cenocepacia, resulting in insights toward development of vaccines and immunotherapeutics to treat or prevent B. cenocepacia infections in the CF patient population.

## MATERIALS AND METHODS

### Bacterial strains.

A list of all Burkholderia cenocepacia strains used in this study can be found in Table 1. B. cenocepacia overnight cultures were grown in Luria-Bertani (LB) medium shaking cultures at 37°C. The BcAU1054 Δ*tecA* and Δ*hcp* deletion mutants were made by allelic exchange (15). For each mutant, ∼500 bp 5′ to and including the first three codons of the gene to delete were fused to ∼500 bp 3′ to and including the last three codons of the gene by splicing by overhang extension PCR. Fusion products were cloned into the allelic exchange vector pEXKm5 (49), and plasmids were conjugated into BcAU1054 using Escherichia coli strain RHO3. Merodiploids were selected on 250 μg/ml kanamycin and grown for 4 h at 37°C with aeration in YT broth (10 g/liter yeast extract, 10 g/liter tryptone), subcultured 1:1,000 on fresh YT broth, and grown overnight at 37°C with aeration. Following overnight growth, cells that had resolved the integrated plasmid were selected on YT agar (1.5% agar) containing 25% sucrose and 100 μg/ml 5-bromo-4-chloro-3-indoxyl-β-d-glucuronide and incubated at 30°C, and deletions were confirmed via PCR and sequencing of the regions spanning the deletions. Complementation of the cloned wild-type (WT) gene or *tecA_C41A_* was done at transposon insertion sites in the B. cenocepacia chromosome (*att*Tn*7* sites) using the pUC18T-mini-Tn7T suite of plasmids (50) and selected by kanamycin resistance. Complementation sequences were cloned into pUC18T-mini-Tn7T-Km containing the constitutive ribosomal S12 subunit gene promoter of Burkholderia thailandensis E264 immediately 5′ to the multiple cloning site (plasmid pUCS12Km, [51]). The *tecA_C41A_* sequence was generated using the Agilent QuikChange II site-directed mutagenesis kit (number 200523). Complementation cassettes were delivered to the BcAU1054 *att*Tn*7* site via triparental mating with E. coli RHO3 strains harboring pUCS12Km-*tecA_WT_*/pUCS12Km-*tecA_C41A_* and the transposase helper plasmid pTNS3, and BcAU1054 exconjugants containing these cassettes were selected on agar containing 250 μg/ml kanamycin. All B. cenocepacia strains used in this study are ampicillin resistant.

### Ethics statement.

Studies requiring mice for isolation of bone barrow and live mice for infections were carried out in accordance with a protocol that adhered to the *Guide for the Care and Use of Laboratory Animals* of the National Institutes of Health (NIH) and was reviewed and approved (approval number 00002184) by the Institutional Animal Care and Use Committee at Dartmouth College. The Dartmouth College animal program is registered with the U.S. Department of Agriculture (USDA) through certificate number 12-R-0001, operates in accordance with Animal Welfare Assurance (NIH/PHS) under assurance number D16-00166 (A3259-01), and is accredited with the Association for Assessment and Accreditation of Laboratory Animal Care International (AAALAC; accreditation number 398). Age-matched, sex-matched, and/or littermate controls were used when appropriate.

### Mouse strains.

Wild-type (WT) C57BL/6J (stock number 000664) mice were purchased from Jackson Laboratories at 7 weeks of age and rested for a week in our mouse facility before infection. Pyrin knockout mice (*Mefv*^−/−^) on the C57BL/6 background were obtained from Jae Chae and Daniel Kastner at the NIH (34) and bred in the mouse facilities at Dartmouth. Mice with the *Cftr^F508del^* mutation on the C57BL/6 background were obtained from Case Western Reserve University’s Cystic Fibrosis Mouse Models Core and bred at Dartmouth. *Mefv*^−/−^ and *Cftr^F508del^* mice were crossed to obtain breeding pairs that were *Cftr^F508del^ Mefv^+/−^* and *Cftr^F508del^ Mefv*^−/−^. The resulting pups were genotyped and used for infections.

### Cell culture.

Bone marrow-derived macrophages (BMDMs) were cultured from bone marrow of mice and cultured as described previously (52, 53). After 7 days of differentiation, the BMDMs were seeded at a density of 0.8 × 10^6^ cells/well in 6-well plates in MGM 10/10 medium containing Dulbecco’s modified Eagle medium (DMEM) plus GlutaMAX (Gibco) containing 10% fetal bovine serum (FBS) (GE), 10% L929 cell-conditioned medium, 1 mM sodium pyruvate (Gibco), 10 mM HEPES (Gibco) and divided into 6-well plates at a density of 0.8 × 10^6^ cells/well in a total volume of 3 ml. The BMDMs were primed with 100 ng/ml O26:B6 Escherichia coli LPS (Sigma) and incubated overnight at 37°C with 5% CO_2_.

### Macrophage infections.

Overnight (16 h) cultures of B. cenocepacia were subcultured 1:100 in fresh LB on infection day and shaken at 37°C until the cultures reached mid-log phase (optical density at 600 nm [OD_600_] = 0.300). Cultures were then pelleted, the LB was removed, and the bacteria were resuspended in phosphate-buffered saline (PBS) to the original volume. The bacterial suspensions were then diluted to an MOI of 20 in warmed, serum-free MGM 10/10. As a positive control for pyrin inflammasome activation, the glucosyltransferase toxin from Clostridium difficile TcdB (List Biological Laboratories, Inc.) was also diluted to 0.1 μg/ml in warmed serum-free MGM 10/10. The BMDMs were washed once in warm 1× PBS, and 3 ml fresh serum-free MGM 10/10 was added with bacteria and TcdB to the appropriate wells. The plates were then centrifuged for 5 min at 1,000 rpm to bring the bacteria and the cells into contact on the bottom of the wells. The plates were incubated at 37°C with 5% CO_2_ for 90 min. Cell supernatants were collected for cytokine ELISAs and lactate dehydrogenase (LDH) assays. The BMDMs were lysed using mammalian protein extraction reagent (M-PER; Thermo Scientific) with added cOmplete, Mini (Roche) protease inhibitor and PhosSTOP (Roche) phosphatase inhibitor.

### Protein analysis by SDS-PAGE and Western blotting.

Five to ten micrograms of protein from the cell lysates was run on 4 to 12% NuPAGE Bis-Tris SDS-PAGE gels (Invitrogen by ThermoFisher Scientific) and transferred to polyvinylidene difluoride membranes (ThermoFisher Scientific) using an iBlot 2 gel transfer device (Life Technologies). Membranes were blocked in 5% nonfat dairy milk and incubated with primary antibodies overnight. The primary antibodies used are rabbit-anti-mouse monoclonal total pyrin antibody (ab195975; abcam), rabbit-anti-mouse monoclonal antibody phospho-serine 205 (ab201784; abcam), rabbit-anti-mouse/human IL-1β (number 12242; Cell Signaling), and rabbit-anti-mouse/human polyclonal β-actin (number 4967; Cell Signaling). Horseradish peroxidase-conjugated anti-rabbit antibody (Jackson Laboratory) was used as a secondary antibody. Proteins were visualized using chemiluminescent detection reagent (GE Healthcare) on an iBright FL1500 (ThermoFisher Scientific).

### IL-1β quantification.

IL-1β in BMDM supernatants was quantified using a murine ELISA kit (MLB00C; R&D Systems) by following the manufacturer’s instructions.

### Tail genotyping.

Genomic DNA was isolated from mouse tail pieces (clipped at time of weaning) by digestion overnight at 55°C in 500 μl lysis buffer (100 mM Tris-HCl, pH 8.5, 5 mM EDTA, 200 mM NaCl, 0.2% SDS, 100 μg/ml proteinase K [VWR]). The supernatant was collected by centrifugation (14,000 rpm, 10 min, room temperature), and DNA was precipitated with 500 μl 100% isopropanol. After centrifugation (14,000 rpm, 10 min, room temperature), the pellets were washed once with 70% ethanol, air dried for 20 min, and dissolved in 200 μl TE buffer at 55°C for 20 min. PCR was performed with the following primers: CTGCCCAGAGAAAGGTGATT, WT F (ATCAAAGAAAATATCATCTTT), *Cftr* WT R (GGACGGTATCATCCCTGAAA), *Cftr* F508del F (ATCAAAGAAAATATCATTGGT), *Cftr* F508del R (ATGGACGGTATCATCCCTGA), internal control F (CTAGGCCACAGAATTGAAAGATCT), internal control R (GTAGGTGGAAATTCTAGCATCATCC), *Mefv* WT F (TGGAAATGGGAGTCCAGAAA), *Mefv* WT R (ACCTACCTGTGGGGTCACTG), *Mefv* KO F (GGGGGAACTTCCTGACTAGG), and *Mefv* KO R (CTGCCCAGAGAAAGGTGATT). To genotype the mouse *Cftr* gene, each tail genomic DNA (gDNA) sample was amplified by PCR in two reactions: one with WT Cftr primers plus internal control and another with F508del Cftr primers plus internal control. To genotype the murine *Mefv* gene, each tail gDNA sample was amplified by PCR in two reactions: one with WT Mefv primers and another with the Mefv KO primers. PCR products were analyzed by agarose gel electrophoresis.

### Mouse infections.

Overnight (16-h) cultures of B. cenocepacia were subcultured 1:100 in fresh LB on infection day and shaken at 37°C until the cultures reached mid-log phase (OD_600_ = 0.300). To prepare the cultures for inoculation, the appropriate volume of culture was centrifuged at 14,000 rpm for 5 min. The LB was removed, the pellets were combined using a small volume of sterile 1× PBS if the original culture volume exceeded the volume of one tube, and the cultures were pelleted again at 14,000 rpm for 5 min. The remaining supernatant was carefully aspirated and the pellet was resuspended in sterile 1× PBS to a final volume for 50 μl/dose. The inoculum was serially diluted, plated on LB plates, grown overnight at 37°C, and counted to ensure the dose was correct for each B. cenocepacia strain used.

Both male and female mice 6 to 12 weeks of age were anesthetized with isoflurane and inoculated via the o.p. route with one 50 μl dose of 5 × 10^7^ CFU of B. cenocepacia or PBS (mock) using a pipette. Mice were weighed immediately following instillation, and weight was monitored at 24-h intervals from time of instillation throughout the experiment. Mice were monitored for survival for 14 days and were checked 3 times a day and euthanized if moribund. At the indicated time points after infection mice were euthanized with CO_2_ or Euthasol with the help of a veterinarian. Tissues including lungs, bronchoalveolar lavage fluid (BALF), liver, and spleen were collected. BALF was collected by inserting a needle into the trachea, removing the lungs and trachea together, and filling the lungs with sterile 1× PBS once with 1 ml and twice after with 500 μl, slowly aspirating and massaging the lungs to recover as much BALF as possible. The cells in the BALF were counted and aliquots of each sample were lysed with either NP-40 (50 mM Tris, pH 8.0, 150 mM NaCl, 1% NP-40) to release intracellular bacteria for subsequent serial dilutions and plating for CFU or M-PER (with protease and phosphatase inhibitors as described above) to generate whole-cell lysates. The protein concentrations of the BALF lysates were quantified, run on SDS-PAGE gels, and used for Western blotting.

To prepare B. cenocepacia antisera, a C57BL/6J mouse was infected as described above, except the route was intranasal and the dose was 1 × 10^8^ CFU of BcAU1054. At day 15 the infection was repeated with a dose of 5 × 10^7^ CFU of BcAU1054. At day 28 the mouse was euthanized and serum was collected and used for IHC along with control serum collected from a naive C57BL/6J mouse.

### Lung fixation, paraffin embedding, and H&E and IHC staining.

Lungs were harvested and the left lungs were used for organ burden (see below) and right lungs were inflated with 10% neutral buffered formalin (Fisher Scientific) and left submerged in formalin for at least 24 h. The right lungs were then dissected into the four different lobes and placed in cassettes submerged in 70% ethanol. The four right lobes for each lung were paraffin embedded and sectioned by the DHMC pathology core. The four lobe sections for each right lung arranged side-by-side on a slide were then stained with hematoxylin and eosin (H&E) for histopathology or were stained to detect specific cell subsets with antibodies suitable for IHC, Ly6G (ab238132; abcam), CD11b (ab133357; abcam), and F4/80 (D2S9R) (number 70076; Cell Signaling), followed by diaminobenzidine (DAB)-conjugated secondary antibody. Alternatively, sections were stained with BcAU1054 antisera and DAB-conjugated secondary antibody to detect the bacteria.

### Microscopy and pathological analysis.

H&E and IHC images of the four lobe sections for each right lung were captured on a Zeiss Axioskop 2 light microscope with a SPOT Insight sCMOS camera (SPOT Advanced Software) maintained by the Dartmouth microscopy core at ×5, ×10, ×20, and ×40 magnification (Plan Neofluar objectives). H&E images of the four lobes from each right lung were blinded and analyzed by Joseph Schwartzman using standard pathological criteria for inflammation and damage.

Semiquantitative H&E scoring and quantitative IHC staining image analysis were performed by HistoWiz. Slides were scanned and the blinded H&E images of the four lobes from each right lung were subjected to semiquantitative scoring of inflammation and damage as described previously (54). Lesion severity was evaluated on a minimal, mild, moderate, and marked scale, and lesion distribution was classified as focal, multifocal, or diffuse (see Table S1 in the supplemental material). Images of the four lung lobe sections from each right lung on a scanned IHC slide were subjected to analysis, and cellular quantification was performed to identify total cells and percentages of DAB-positive cells classified by weak, medium, or intense stain. Values were normalized by H-score, calculated as 1× (% weak stain) + 2× (% medium stain) + 3× (% intense stain).

### Organ burden.

Organs were collected in stomacher bags, weighed, and placed on ice. Five milliliters of sterile 1× PBS was added to full (left and right) lungs and livers, and 2.5 ml of sterile 1× PBS was added to spleens and left lungs. Organs were homogenized by rolling out by hand and then by use of a Stomacher 80 Biomaster (Seward) on high setting for 2 min. For lungs this homogenization method was done twice to increase homogenization of the tissue. The homogenates were then serially diluted in sterile, 1× PBS and plated on LB or LB plates with 100 μg/ml ampicillin. The plates were incubated at 37°C, and the colonies were counted at least 24 h later. Data are displayed as number of CFU per gram of tissue.

### Statistical analysis.

GraphPad Prism was used to perform statistical analyses. IL-1β ELISA data were analyzed by one-way or two-way analysis of variance (ANOVA; Tukey’s multiple-comparison test) or Mann-Whitney test. Analysis of the weight loss data was performed by Thomas H. Hampton in the R statistical programming language including ggplot2. Asymptotic Wilcoxon-Mann-Whitney tests were performed using the coin package. Weight loss data were also analyzed using Welch’s *t* test. Survival data were analyzed using the log-rank (Mantel-Cox) test. CFU data were analyzed by Mann-Whitney test. Additional details for each experiment can be found in the figure legends.

## References

[1] Loutet SA, Valvano MA. 2010. A decade of Burkholderia cenocepacia virulence determinant research. Infect Immun 78:4088–4100. doi:10.1128/IAI.00212-10. 20643851 PMC2950345

[2] Mahenthiralingam E, Urban TA, Goldberg JB. 2005. The multifarious, multireplicon Burkholderia cepacia complex. Nat Rev Microbiol 3:144–156. doi:10.1038/nrmicro1085. 15643431

[3] Hauser AR, Jain M, Bar-Meir M, McColley SA. 2011. Clinical significance of microbial infection and adaptation in cystic fibrosis. Clin Microbiol Rev 24:29–70. doi:10.1128/CMR.00036-10. 21233507 PMC3021203

[4] Ratjen F, Bell SC, Rowe SM, Goss CH, Quittner AL, Bush A. 2015. Cystic fibrosis. Nat Rev Dis Primers 1:15010. doi:10.1038/nrdp.2015.10. 27189798 PMC7041544

[5] Wang XR, Li C. 2014. Decoding F508del misfolding in cystic fibrosis. Biomolecules 4:498–509. doi:10.3390/biom4020498. 24970227 PMC4101494

[6] Yoshimura K, Nakamura H, Trapnell BC, Chu CS, Dalemans W, Pavirani A, Lecocq JP, Crystal RG. 1991. Expression of the cystic fibrosis transmembrane conductance regulator gene in cells of non-epithelial origin. Nucleic Acids Res 19:5417–5423. doi:10.1093/nar/19.19.5417. 1717947 PMC328907

[7] Bonfield TL, Hodges CA, Cotton CU, Drumm ML. 2012. Absence of the cystic fibrosis transmembrane regulator (Cftr) from myeloid-derived cells slows resolution of inflammation and infection. J Leukoc Biol 92:1111–1122. doi:10.1189/jlb.0412188. 22859830 PMC3476241

[8] Ng HP, Valentine VG, Wang G. 2016. CFTR targeting during activation of human neutrophils. J Leukoc Biol 100:1413–1424. doi:10.1189/jlb.4A0316-130RR. 27406994

[9] Ng HP, Zhou Y, Song K, Hodges CA, Drumm ML, Wang G. 2014. Neutrophil-mediated phagocytic host defense defect in myeloid Cftr-inactivated mice. PLoS One 9:e106813. doi:10.1371/journal.pone.0106813. 25184794 PMC4153692

[10] Lee AH, Flibotte S, Sinha S, Paiero A, Ehrlich RL, Balashov S, Ehrlich GD, Zlosnik JE, Mell JC, Nislow C. 2017. Phenotypic diversity and genotypic flexibility of Burkholderia cenocepacia during long-term chronic infection of cystic fibrosis lungs. Genome Res 27:650–662. doi:10.1101/gr.213363.116. 28325850 PMC5378182

[11] Ganesan S, Sajjan US. 2011. Host evasion by Burkholderia cenocepacia. Front Cell Infect Microbiol 1:25. 22919590 10.3389/fcimb.2011.00025 PMC3417383

[12] Schwab U, Abdullah LH, Perlmutt OS, Albert D, Davis CW, Arnold RR, Yankaskas JR, Gilligan P, Neubauer H, Randell SH, Boucher RC. 2014. Localization of Burkholderia cepacia complex bacteria in cystic fibrosis lungs and interactions with Pseudomonas aeruginosa in hypoxic mucus. Infect Immun 82:4729–4745. doi:10.1128/IAI.01876-14. 25156735 PMC4249344

[13] Sousa SA, Ulrich M, Bragonzi A, Burke M, Worlitzsch D, Leitao JH, Meisner C, Eberl L, Sa-Correia I, Doring G. 2007. Virulence of Burkholderia cepacia complex strains in gp91phox-/- mice. Cell Microbiol 9:2817–2825. doi:10.1111/j.1462-5822.2007.00998.x. 17627623

[14] Sajjan U, Thanassoulis G, Cherapanov V, Lu A, Sjolin C, Steer B, Wu YJ, Rotstein OD, Kent G, McKerlie C, Forstner J, Downey GP. 2001. Enhanced susceptibility to pulmonary infection with Burkholderia cepacia in Cftr(-/-) mice. Infect Immun 69:5138–5150. doi:10.1128/IAI.69.8.5138-5150.2001. 11447196 PMC98610

[15] Perault AI, Chandler CE, Rasko DA, Ernst RK, Wolfgang MC, Cotter PA. 2020. Host adaptation predisposes Pseudomonas aeruginosa to type VI secretion system-mediated predation by the Burkholderia cepacia complex. Cell Host Microbe 28:534–547. doi:10.1016/j.chom.2020.06.019. 32755549 PMC7554260

[16] Spiewak HL, Shastri S, Zhang L, Schwager S, Eberl L, Vergunst AC, Thomas MS. 2019. Burkholderia cenocepacia utilizes a type VI secretion system for bacterial competition. Microbiologyopen doi:10.1002/mbo3.774. PMC6612558 30628184

[17] Valvano MA. 2015. Intracellular survival of Burkholderia cepacia complex in phagocytic cells. Can J Microbiol 61:607–615. doi:10.1139/cjm-2015-0316. 26220706

[18] Hunt TA, Kooi C, Sokol PA, Valvano MA. 2004. Identification of Burkholderia cenocepacia genes required for bacterial survival in vivo. Infect Immun 72:4010–4022. doi:10.1128/IAI.72.7.4010-4022.2004. 15213146 PMC427415

[19] Gavrilin MA, Abdelaziz DH, Mostafa M, Abdulrahman BA, Grandhi J, Akhter A, Abu Khweek A, Aubert DF, Valvano MA, Wewers MD, Amer AO. 2012. Activation of the pyrin inflammasome by intracellular Burkholderia cenocepacia. J Immunol 188:3469–3477. doi:10.4049/jimmunol.1102272. 22368275 PMC3482472

[20] Kovacs SB, Miao EA. 2017. Gasdermins: effectors of pyroptosis. Trends Cell Biol 27:673–684. doi:10.1016/j.tcb.2017.05.005. 28619472 PMC5565696

[21] Shi J, Gao W, Shao F. 2017. Pyroptosis: gasdermin-mediated programmed necrotic cell death. Trends Biochem Sci 42:245–254. doi:10.1016/j.tibs.2016.10.004. 27932073

[22] Xu H, Yang J, Gao W, Li L, Li P, Zhang L, Gong YN, Peng X, Xi JJ, Chen S, Wang F, Shao F. 2014. Innate immune sensing of bacterial modifications of Rho GTPases by the Pyrin inflammasome. Nature 513:237–241. doi:10.1038/nature13449. 24919149

[23] de Zoete MR, Flavell RA. 2014. Detecting “different”: pyrin senses modified GTPases. Cell Res 24:1286–1287. doi:10.1038/cr.2014.101. 25091449 PMC4220150

[24] Loeven NA, Medici NP, Bliska JB. 2020. The pyrin inflammasome in host-microbe interactions. Curr Opin Microbiol 54:77–86. doi:10.1016/j.mib.2020.01.005. 32120337 PMC7247927

[25] Schnappauf O, Chae JJ, Kastner DL, Aksentijevich I. 2019. The pyrin inflammasome in health and disease. Front Immunol 10:1745. doi:10.3389/fimmu.2019.01745. 31456795 PMC6698799

[26] Aubert DF, Xu H, Yang J, Shi X, Gao W, Li L, Bisaro F, Chen S, Valvano MA, Shao F. 2016. A Burkholderia type VI effector deamidates Rho GTPases to activate the pyrin inflammasome and trigger inflammation. Cell Host Microbe 19:664–674. doi:10.1016/j.chom.2016.04.004. 27133449

[27] Abdulrahman BA, Khweek AA, Akhter A, Caution K, Kotrange S, Abdelaziz DH, Newland C, Rosales-Reyes R, Kopp B, McCoy K, Montione R, Schlesinger LS, Gavrilin MA, Wewers MD, Valvano MA, Amer AO. 2011. Autophagy stimulation by rapamycin suppresses lung inflammation and infection by Burkholderia cenocepacia in a model of cystic fibrosis. Autophagy 7:1359–1370. doi:10.4161/auto.7.11.17660. 21997369 PMC3359483

[28] Robledo-Avila FH, Ruiz-Rosado JD, Brockman KL, Kopp BT, Amer AO, McCoy K, Bakaletz LO, Partida-Sanchez S. 2018. Dysregulated calcium homeostasis in cystic fibrosis neutrophils leads to deficient antimicrobial responses. J Immunol 201:2016–2027. doi:10.4049/jimmunol.1800076. 30120123 PMC6143431

[29] Park YH, Wood G, Kastner DL, Chae JJ. 2016. Pyrin inflammasome activation and RhoA signaling in the autoinflammatory diseases FMF and HIDS. Nat Immunol 17:914–921. doi:10.1038/ni.3457. 27270401 PMC4955684

[30] Gao W, Yang J, Liu W, Wang Y, Shao F. 2016. Site-specific phosphorylation and microtubule dynamics control Pyrin inflammasome activation. Proc Natl Acad Sci USA 113:E4857–E4866. doi:10.1073/pnas.1601700113. 27482109 PMC4995971

[31] Izaguirre-Anariba DE, Sivapalan V. 2020. Chryseobacterium indologenes, an emerging bacteria: a case report and review of literature. Cureus 12:e6720. doi:10.7759/cureus.6720. 32104641 PMC7032597

[32] Hagiya H, Ohnishi K, Maki M, Watanabe N, Murase T. 2013. Clinical characteristics of Ochrobactrum anthropi bacteremia. J Clin Microbiol 51:1330–1333. doi:10.1128/JCM.03238-12. 23363833 PMC3666774

[33] Hachani A, Wood TE, Filloux A. 2016. Type VI secretion and anti-host effectors. Curr Opin Microbiol 29:81–93. doi:10.1016/j.mib.2015.11.006. 26722980

[34] Chae JJ, Cho YH, Lee GS, Cheng J, Liu PP, Feigenbaum L, Katz SI, Kastner DL. 2011. Gain-of-function Pyrin mutations induce NLRP3 protein-independent interleukin-1beta activation and severe autoinflammation in mice. Immunity 34:755–768. doi:10.1016/j.immuni.2011.02.020. 21600797 PMC3129608

[35] Rosales-Reyes R, Skeldon AM, Aubert DF, Valvano MA. 2012. The Type VI secretion system of Burkholderia cenocepacia affects multiple Rho family GTPases disrupting the actin cytoskeleton and the assembly of NADPH oxidase complex in macrophages. Cell Microbiol 14:255–273. doi:10.1111/j.1462-5822.2011.01716.x. 22023353

[36] Estfanous S, Krause K, Anne MNK, Eltobgy M, Caution K, Abu Khweek A, Hamilton K, Badr A, Daily K, Carafice C, Baetzhold D, Zhang X, Li T, Wen H, Gavrilin MA, Haffez H, Soror S, Amer AO. 2021. Gasdermin D restricts Burkholderia cenocepacia infection in vitro and in vivo. Sci Rep 11:855. doi:10.1038/s41598-020-79201-5. 33441602 PMC7807041

[37] Krause K, Caution K, Badr A, Hamilton K, Saleh A, Patel K, Seveau S, Hall-Stoodley L, Hegazi R, Zhang X, Gavrilin MA, Amer AO. 2018. CASP4/caspase-11 promotes autophagosome formation in response to bacterial infection. Autophagy 14:1928–1942. doi:10.1080/15548627.2018.1491494. 30165781 PMC6152495

[38] Flannagan RS, Jaumouille V, Huynh KK, Plumb JD, Downey GP, Valvano MA, Grinstein S. 2012. Burkholderia cenocepacia disrupts host cell actin cytoskeleton by inactivating Rac and Cdc42. Cell Microbiol 14:239–254. doi:10.1111/j.1462-5822.2011.01715.x. 22023324

[39] Huynh KK, Plumb JD, Downey GP, Valvano MA, Grinstein S. 2010. Inactivation of macrophage Rab7 by Burkholderia cenocepacia. J Innate Immun 2:522–533. doi:10.1159/000319864. 20829607 PMC2982851

[40] Lamothe J, Huynh KK, Grinstein S, Valvano MA. 2007. Intracellular survival of Burkholderia cenocepacia in macrophages is associated with a delay in the maturation of bacteria-containing vacuoles. Cell Microbiol 9:40–53. doi:10.1111/j.1462-5822.2006.00766.x. 16869828

[41] Walpole GFW, Plumb JD, Chung D, Tang B, Boulay B, Osborne DG, Piotrowski JT, Catz SD, Billadeau DD, Grinstein S, Jaumouille V. 2020. Inactivation of Rho GTPases by Burkholderia cenocepacia induces a WASH-mediated actin polymerization that delays phagosome maturation. Cell Rep 31:107721. doi:10.1016/j.celrep.2020.107721. 32492429 PMC7315377

[42] Keith KE, Hynes DW, Sholdice JE, Valvano MA. 2009. Delayed association of the NADPH oxidase complex with macrophage vacuoles containing the opportunistic pathogen Burkholderia cenocepacia. Microbiology (Reading) 155:1004–1015. doi:10.1099/mic.0.026781-0. 19332803

[43] Assani K, Shrestha CL, Robledo-Avila F, Rajaram MV, Partida-Sanchez S, Schlesinger LS, Kopp BT. 2017. Human cystic fibrosis macrophages have defective calcium-dependent protein kinase C activation of the NADPH oxidase, an effect augmented by Burkholderia cenocepacia. J Immunol 198:1985–1994. doi:10.4049/jimmunol.1502609. 28093527 PMC5322234

[44] Heung LJ, Hohl TM. 2019. Inflammatory monocytes are detrimental to the host immune response during acute infection with Cryptococcus neoformans. PLoS Pathog 15:e1007627. doi:10.1371/journal.ppat.1007627. 30897162 PMC6428256

[45] Warny M, Keates AC, Keates S, Castagliuolo I, Zacks JK, Aboudola S, Qamar A, Pothoulakis C, LaMont JT, Kelly CP. 2000. p38 MAP kinase activation by Clostridium difficile toxin A mediates monocyte necrosis, IL-8 production, and enteritis. J Clin Invest 105:1147–1156. doi:10.1172/JCI7545. 10772660 PMC300827

[46] Bobo LD, El Feghaly RE, Chen YS, Dubberke ER, Han Z, Baker AH, Li J, Burnham CA, Haslam DB. 2013. MAPK-activated protein kinase 2 contributes to Clostridium difficile-associated inflammation. Infect Immun 81:713–722. doi:10.1128/IAI.00186-12. 23264053 PMC3584893

[47] Nakamura H, Yoshimura K, McElvaney NG, Crystal RG. 1992. Neutrophil elastase in respiratory epithelial lining fluid of individuals with cystic fibrosis induces interleukin-8 gene expression in a human bronchial epithelial cell line. J Clin Invest 89:1478–1484. doi:10.1172/JCI115738. 1569186 PMC443018

[48] Zaman MM, Gelrud A, Junaidi O, Regan MM, Warny M, Shea JC, Kelly C, O'Sullivan BP, Freedman SD. 2004. Interleukin 8 secretion from monocytes of subjects heterozygous for the deltaF508 cystic fibrosis transmembrane conductance regulator gene mutation is altered. Clin Vaccine Immunol 11:819–824. doi:10.1128/CDLI.11.5.819-824.2004. PMC515258 15358638

[49] Lopez CM, Rholl DA, Trunck LA, Schweizer HP. 2009. Versatile dual-technology system for markerless allele replacement in Burkholderia pseudomallei. Appl Environ Microbiol 75:6496–6503. doi:10.1128/AEM.01669-09. 19700544 PMC2765137

[50] Choi KH, Gaynor JB, White KG, Lopez C, Bosio CM, Karkhoff-Schweizer RR, Schweizer HP. 2005. A Tn7-based broad-range bacterial cloning and expression system. Nat Methods 2:443–448. doi:10.1038/nmeth765. 15908923

[51] Anderson MS, Garcia EC, Cotter PA. 2012. The Burkholderia bcpAIOB genes define unique classes of two-partner secretion and contact dependent growth inhibition systems. PLoS Genet 8:e1002877. doi:10.1371/journal.pgen.1002877. 22912595 PMC3415462

[52] Brodsky IE, Palm NW, Sadanand S, Ryndak MB, Sutterwala FS, Flavell RA, Bliska JB, Medzhitov R. 2010. A Yersinia effector protein promotes virulence by preventing inflammasome recognition of the type III secretion system. Cell Host Microbe 7:376–387. doi:10.1016/j.chom.2010.04.009. 20478539 PMC2883865

[53] Chung LK, Park YH, Zheng Y, Brodsky IE, Hearing P, Kastner DL, Chae JJ, Bliska JB. 2016. The Yersinia virulence factor YopM hijacks host kinases to inhibit type III effector-triggered activation of the pyrin inflammasome. Cell Host Microbe 20:296–306. doi:10.1016/j.chom.2016.07.018. 27569559 PMC5025386

[54] Fukushi M, Ito T, Oka T, Kitazawa T, Miyoshi-Akiyama T, Kirikae T, Yamashita M, Kudo K. 2011. Serial histopathological examination of the lungs of mice infected with influenza A virus PR8 strain. PLoS One 6:e21207. doi:10.1371/journal.pone.0021207. 21701593 PMC3118813

