# Supplementary figures and images for: The Burkholderia cenocepacia Type VI Secretion System Effector TecA Is a Virulence Factor in Mouse Models of Lung Infection
Source: mBio. 2021 Sep 28;12(5):e02098-21. doi: 10.1128/mBio.02098-21 (PMC8546862; doi:10.1128/mBio.02098-21)

**FIG S1.**

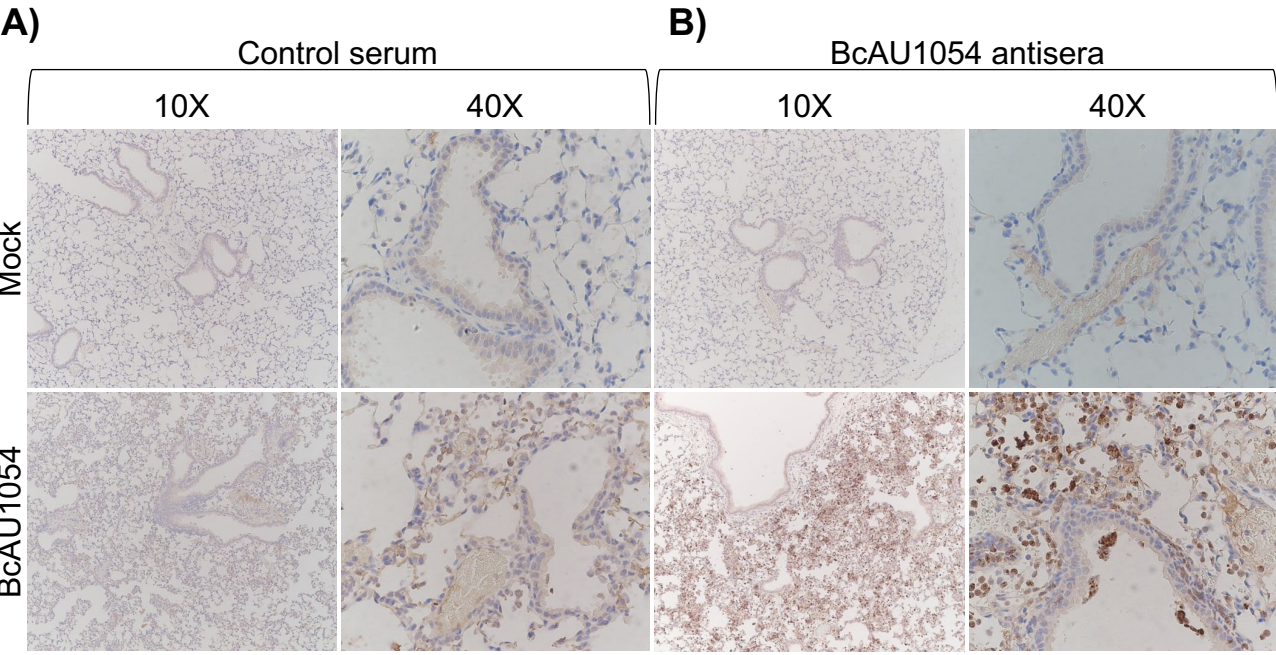

Supplement: FIG S1 [file mbio.02098-21-sf001.pdf]

FIG S2.

A)

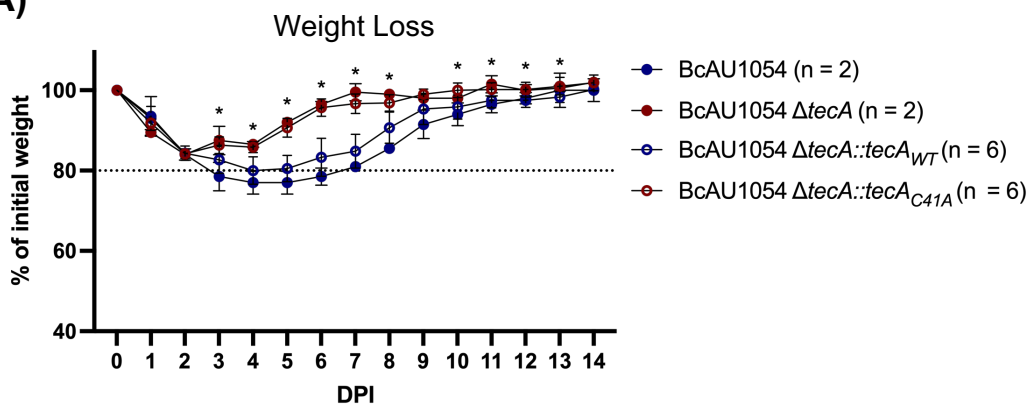

Supplement: FIG S2 [file mbio.02098-21-sf002.pdf]

**FIG S4.**

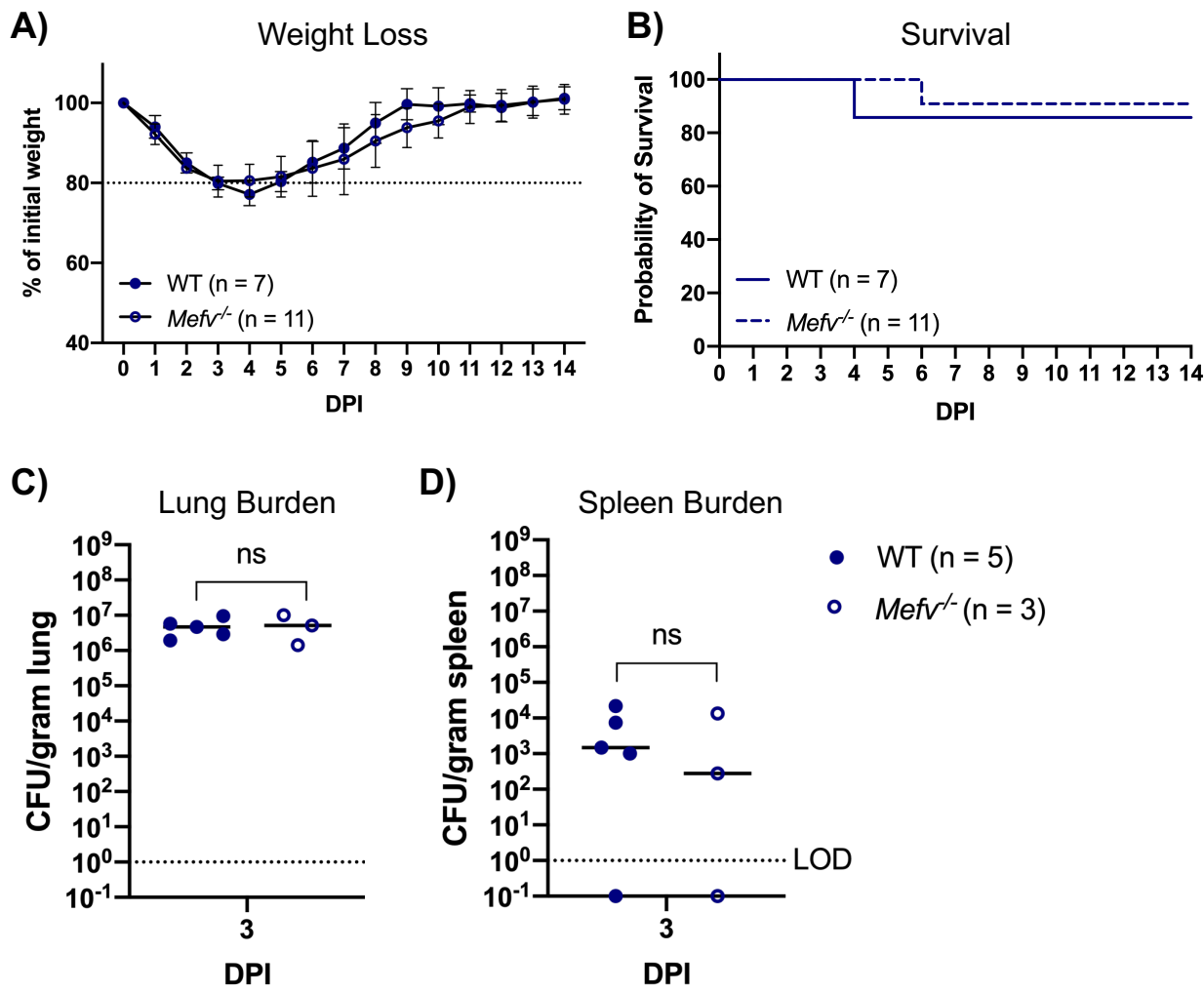

Supplement: FIG S4 [file mbio.02098-21-sf004.pdf]

# FIG S3.

A)

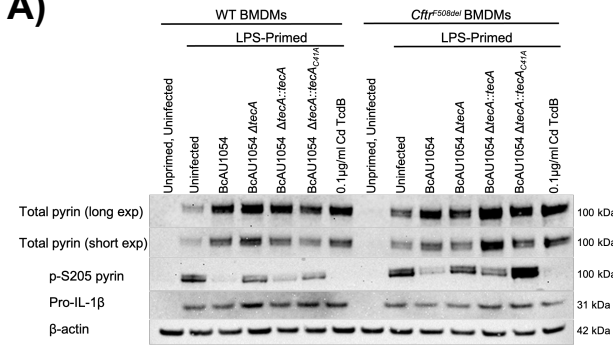

B)

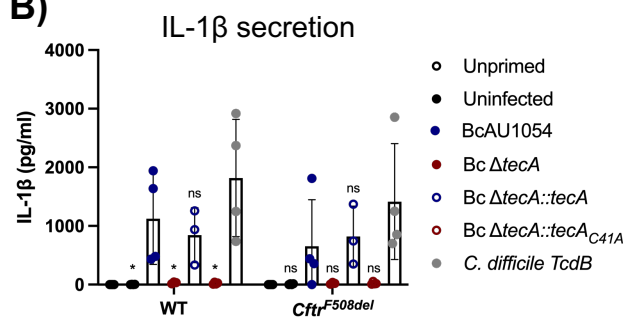

C)

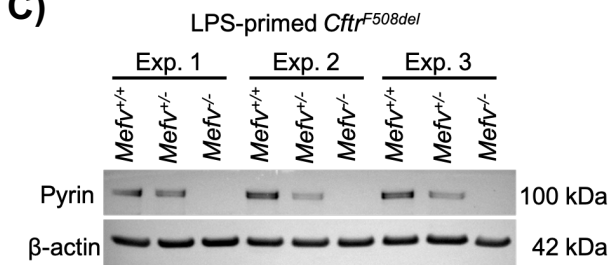

D)

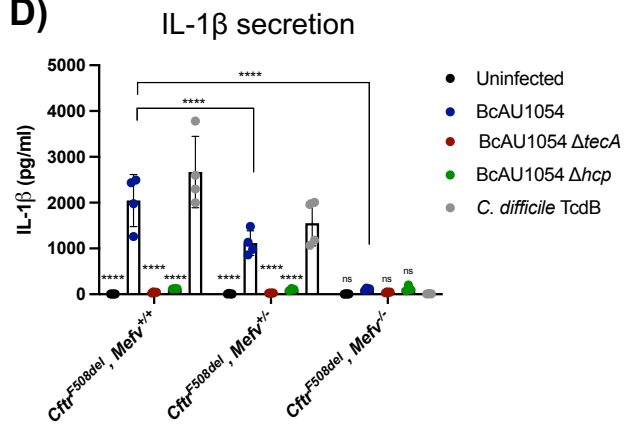

E)

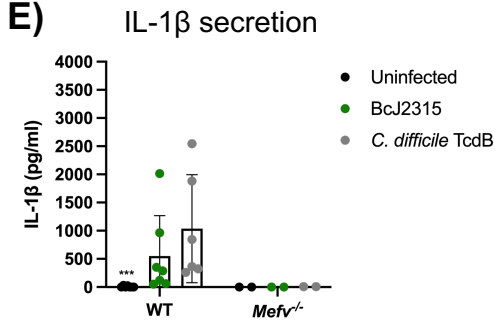

Supplement: FIG S3 [file mbio.02098-21-sf003.pdf]

**FIG S5.**

**A)**

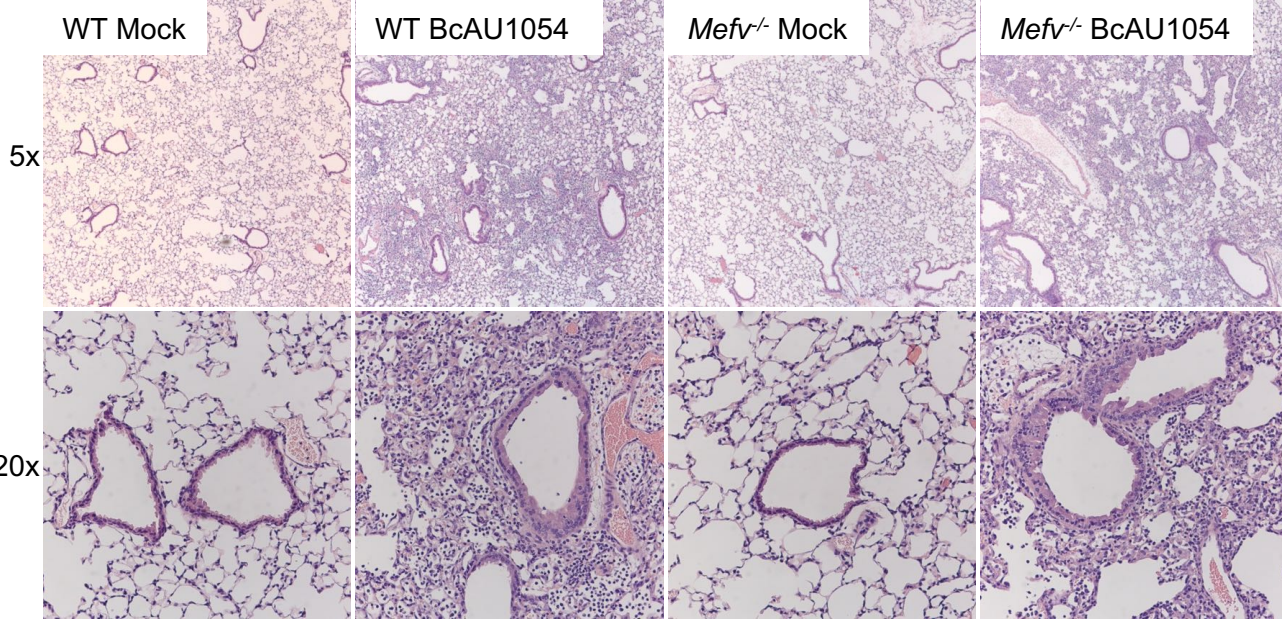

**B)**

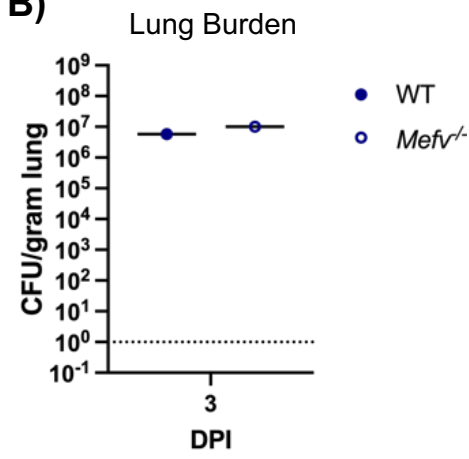

Supplement: FIG S5 [file mbio.02098-21-sf005.pdf]

**FIG S6.**

**A)**

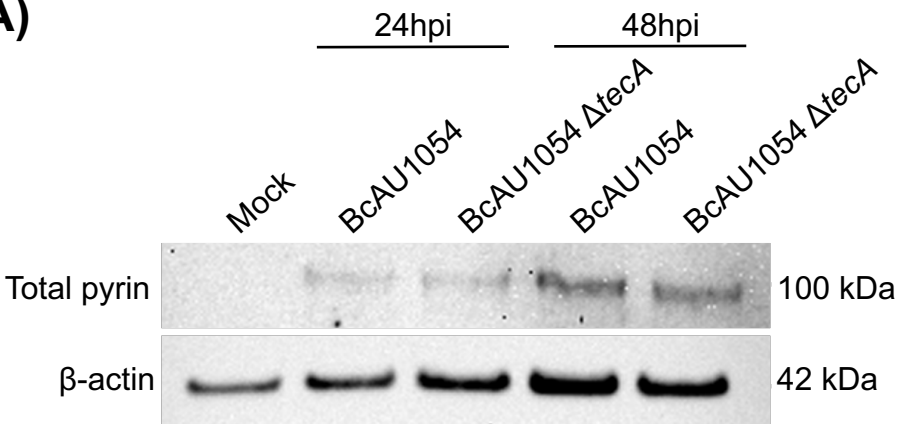

Supplement: FIG S6 [file mbio.02098-21-sf006.pdf]

FIG S7.

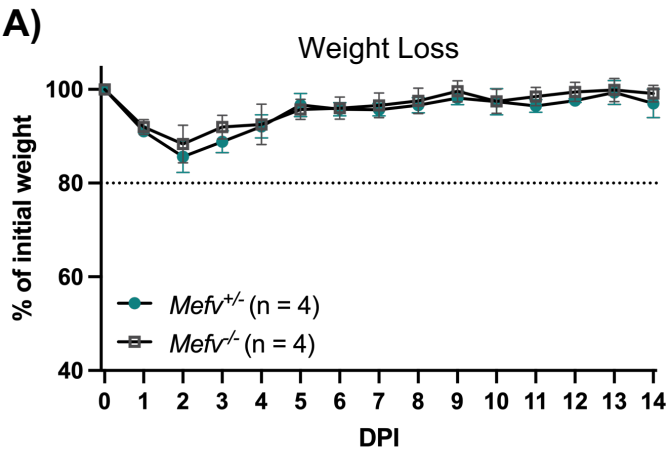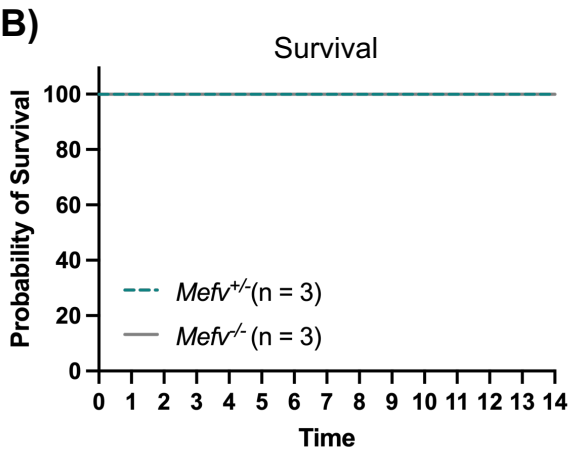

Supplement: FIG S7 [file mbio.02098-21-sf007.pdf]

**FIG S8.**

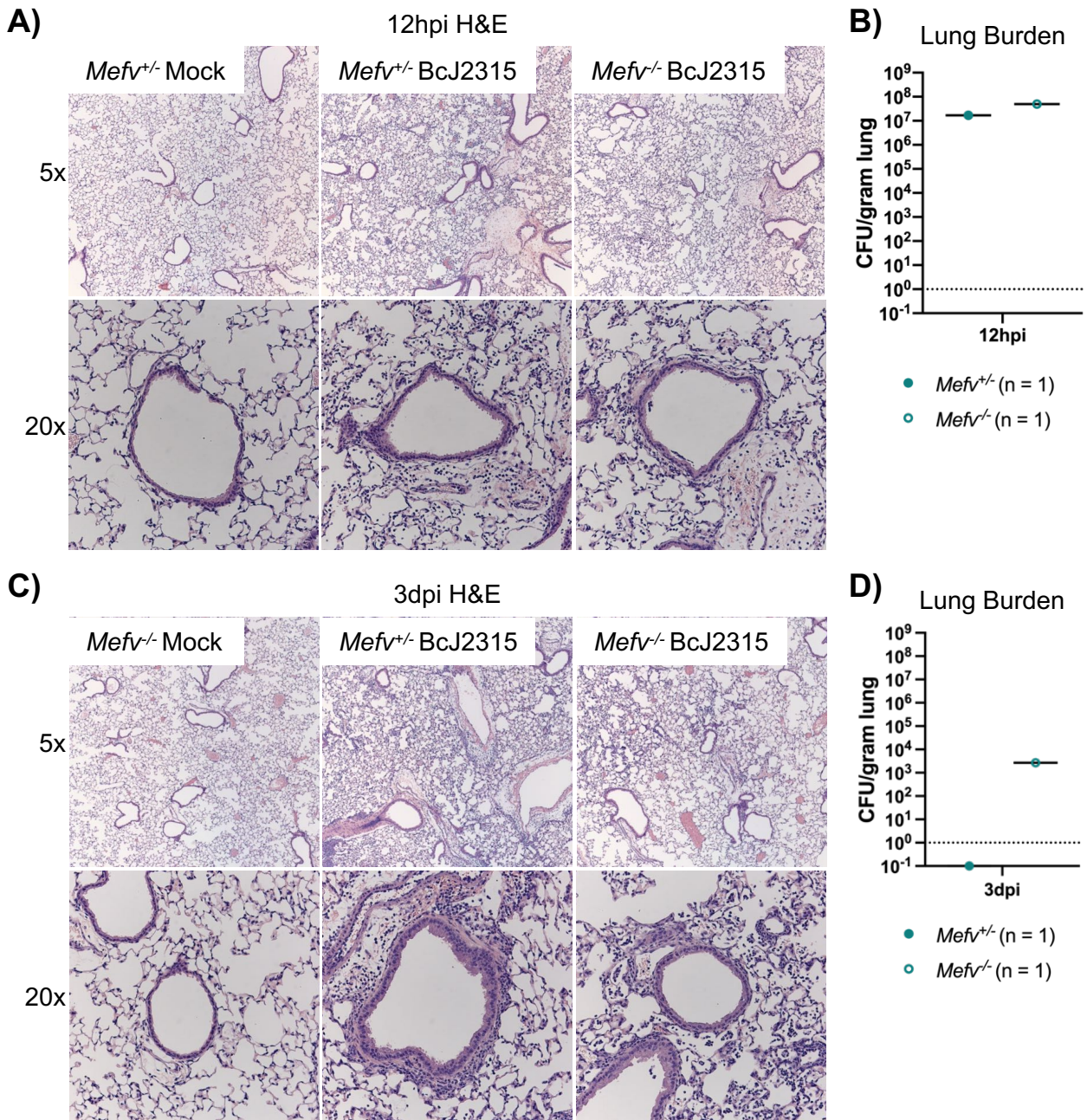

Supplement: FIG S8 [file mbio.02098-21-sf008.pdf]
