# Supplementary material for: The Burkholderia cenocepacia Type VI Secretion System Effector TecA Is a Virulence Factor in Mouse Models of Lung Infection
Source: mBio. 2021 Sep 28;12(5):e02098-21. doi: 10.1128/mBio.02098-21 (PMC8546862; doi:10.1128/mBio.02098-21)
Supplement: TABLE S1 [file mbio.02098-21-st001.pdf]

**Table S1.**

| Summary of semi-quantitative scoring of lung inflammation and damage. | | | |
|---|---|---|---|
| Timepoint | Condition | Bronchioles | Alveoli |
| **12hpi** | **Mock** | Normal | Normal |
| | **BcAU1054** | Intraluminal neutrophilic and mononuclear cell infiltrate, mild, multifocal | Neutrophilic and histiocytic inflammatory infiltrate, multifocal, mild<br>Alveolar collapse: mild, multifocal<br>Alveolar septal thickening: mild, multifocal |
| | **Bc AU1054 Δ*tecA*** | Intraluminal neutrophilic and mononuclear cell infiltrate, moderate, multifocal | Neutrophilic and histiocytic inflammatory infiltrate, moderate, multifocal<br>Alveolar collapse: mild, multifocal<br>Alveolar septal thickening: mild, multifocal |
| **3dpi** | **Mock** | Normal | Normal |
| | **BcAU1054** | Intraluminal neutrophilic and mononuclear cell infiltrate, mild, multifocal | Neutrophilic and histiocytic inflammatory infiltrate, marked, multifocal<br>Alveolar collapse: moderate, multifocal<br>Alveolar septal thickening: moderate, multifocal |
| | **Bc AU1054 Δ*tecA*** | Intraluminal neutrophilic and mononuclear cell infiltrate, mild, multifocal | Neutrophilic and histiocytic inflammatory infiltrate, marked, multifocal<br>Alveolar collapse: marked, multifocal<br>Alveolar septal thickening: marked, multifocal |
